# MEOX2-mediated regulation of Cathepsin S promotes cell proliferation and motility in glioma

**DOI:** 10.1038/s41419-022-04845-2

**Published:** 2022-04-18

**Authors:** Ji Wang, Yanming Chen, Qing Wang, Hui Xu, Chunwang Wu, Qianqian Jiang, Guoqing Wu, Honglong Zhou, Zongyu Xiao, Ying Chen, Tan Zhang, Qing Lan

**Affiliations:** 1 Department of Neurosurgery, The Second Affiliated Hospital of Soochow University, Suzhou, 215004 China; 2 Jiangsu Key Laboratory of Neuropsychiatric Disease, Institute of Neuroscience, Soochow University, Suzhou, 215123 China; 3 Department of Neurosurgery, The Second Affiliated Hospital of Nanchang University, Nanchang, 330006 China; 4 Department of Neurosurgery, Dushu Lake Hospital, Soochow University, Suzhou, 215124 China

**Keywords:** CNS cancer, Preclinical research

## Abstract

Nuclear transcription factor Mesenchyme Homeobox 2 (MEOX2) is a homeobox gene that is originally discovered to suppress the growth of vascular smooth muscle and endothelial cells. However, whether or not it is connected to cancer is yet unknown. Here, we report that MEOX2 functions as a tumor-initiating element in glioma. Bioinformatic analyses of public databases and investigation of MEOX2 expression in patients with glioma demonstrated that MEOX2 was abundant at both mRNA and protein levels in glioma. MEOX2 expression was shown to be inversely linked with the prognosis of glioma patients. MEOX2 inhibition changed the morphology of glioma cells, inhibited cell proliferation and motility, whereas had no effect on cell apoptosis. Besides, silencing MEOX2 also hampered the epithelial-mesenchymal transition (EMT), focal adhesion formation, and F-actin assembly. Overexpression of MEOX2 exhibited opposite effects. Importantly, RNA-sequencing, ChIP-qPCR assay, and luciferase reporter assay revealed Cathepsin S (CTSS) as a novel transcriptional target of MEOX2 in glioma cells. Consistently, MEOX2 causes glioma tumor development in mice and greatly lowers the survival period of tumor-bearing mice. Our findings indicate that MEOX2 promotes tumorigenesis and progression of glioma partially through the regulation of CTSS. Targeting MEOX2-CTSS axis might be a promising alternative for the treatment of glioma.

## Introduction

Glioma is infamous as the most common and malignant primary tumor of central nervous system [1]. Glioblastoma (GBM) is the highest-grade glioma, as well as the most form of glioma in adults (57.7% of glioma). Despite current advances in multimodality therapy for GBM, the patients remain with an undesirable prognosis and a poor median survival of only 14.6 months [2]. Tumorigenesis and highly aggressiveness are two pivotal pathological characteristics for glioma development. Though various genetic and epigenetic regulations synergistically contribute to the progression of glioma, the key element triggering or inhibiting these processes is still largely unknown. Therefore, for improving the early diagnosis and therapeutic effect of glioma, a greater understanding and illustration of the molecular events involved in the initiation and progression of glioma are important research goals.

Mesenchyme Homeobox 2 (MEOX2, also called GAX), which belongs to the homeobox gene family, was discovered as a nuclear transcription factor and was also identified as an inhibitory factor of the vascular smooth muscle cell and vascular endothelial cell proliferation [3–5]. It is reported that overexpression MEOX2 could suppress vascular endothelial cell growth and migration by cyclin-dependent kinase inhibitor up-regulation and NF-κB pathway inactivation [6–8]. Besides, MEOX2 is reported as an oncogene or tumor suppressor gene in various cancers. In lung cancer, overexpression of MEOX2 has been associated with chemoresistance and prognosis, whereas patients with lower MEOX2 expression had shorter survival times in hepatocellular carcinoma and larynx carcinoma [9–12]. Attractively, recent publications described MEOX2 significantly upregulation in glioma and predicted MEOX2 as a prognostic biomarker of glioma [13, 14]. However, its specific role and the underlying mechanisms in glioma remain poorly understood.

In this study, we demonstrated that MEOX2 contributes to cell proliferation and motility in glioma. We identified Cathepsin S (CTSS), which is from the Cathepsin family of cysteine proteases, has been implicated in an important role for tumor development and progression [15], as a MEOX2 downstream target. The regulatory mechanism and biological effects of MEOX2 and CTSS were also determined. Our findings indicate that MEOX2-CTSS axis has the potential to be prognostic markers and therapeutic targets for glioma.

## Materials and methods

### Clinical samples

From February 2017 to September 2020, a total of 39 human brain glioma tissues and 5 normal brain tissues (NBT) were enrolled in this study, which was taken during glioma surgery at the Second Affiliated Hospital of Soochow University (Suzhou, Jiangsu, China). The Ethical Committee of the Second Affiliated Hospital of Soochow University authorized the collection of all clinical specimens, and each patient or legal guardian provided written informed permission.

### Cell culture

Human GBM cell lines (U87, U118, U251, SNB19, LN229), Normal Human Astrocytes (NHA), and glioma stem cell (GSC) lines (GSC23, GSC11, GSC91) were provided by Dr. Li Ming (Department of Neurosurgery, University of Minnesota). The NHA and GBM cell lines were grown in Dulbecco’s Modified Eagle’s Medium (DMEM) with 10% fetal bovine serum (FBS). GSC were cultured in DMEM/F12 (1:1) medium with B27, EGF (20 ng/mL), and bFGF (20 ng/mL) supplementation. All of the cells were kept at 37 °C in a humidified condition with 5% CO_2_.

### Transient transfection with siRNA

Two siRNAs targeting MEOX2 (siMEOX2 #1 sense sequence: 5’- GCATTTACCAAAGAGCAAA-3’, siMEOX2 #2 sense sequence: 5’- GGTAAAGGGTGGACAGCAA-3’) and Scramble siRNA (sense sequence: 5’- UUCUCCGAACGUGUCACGUTT-3’) were generated by GenePharma (Suzhou, China). CTSS siRNA (sc-29940) and Scramble siRNA (sc-37007) were obtained from Santa Cruz Biotechnology (Santa Cruz, CA, USA). The cells were seeded in a 6-well plate and allowed to reach a confluence of 60–70% on the second day for transfection. According to the manufacturer’s instructions, the siRNAs were transfected into cells using Lipofectamine 3000 (Invitrogen, USA). Target gene expression was evaluated by qRT-PCR and immunoblot after 48 h of transfection.

### Generation of stable cells

Lentiviral expression MEOX2-shRNA, Scramble-shRNA, MEOX2, CT SS, and Empty Vector (EV) were constructed from Genechem (Shanghai, China). The shMEOX2 sequence was designed according to the siMEOX2 targeting sequence #2. GSC23 cells were utilized to create a stable MEOX2 knockdown cell line, whereas U87 and GSC91 cells were used to create stable MEOX2 overexpression cell lines. GSC23 or GSC91 cell spheres were trypsinized into individual cells and then plated on 24-well Matrigel pre-coated plates, while U87 cells were directly plated in 24-well plates. Lentiviruses transfection was performed using polybrene according to the manufacturer’s instructions. After 48 h of transfection, the cells were screened with 5 µg/mL puromycin (MCE, USA) for 2 weeks to obtain a stable cell line, and qRT-PCR and immunoblot were used to detect transfection efficiency.

### CCK-8 assay

The Cell Counting Kit-8 (CCK-8) test was carried out to assess cell viability using a CCK-8 kit (Dojindo, Japan). The cells were grown in a growth medium in 5 duplicate wells (2 × 10^3^ cells/well) of a 96-well plate for 0, 24, 48, 72, 96, or 120 h, respectively. Then, at a predetermined time, the medium was replaced with DMEM/10% FBS containing 10% CCK-8 solution at 37 °C for 1 h in the dark. The plate was read on a microplate reader (TECAN, Switzerland) at 450 nm wavelength.

### EdU assay

The EdU assay was also used to evaluate cell proliferation by an EdU kit (RiboBio, Guangzhou, China) according to the manufacturer’s instructions. In brief, the cells were grown in 35 mm glass-bottom dishes (Cellvis, USA) and incubated with EdU solution (medium: EdU = 1000: 1) at 37 °C for 4 h. The cells were then fixed with 4% paraformaldehyde (PFA) and stained with Apollo Dye solution at RT for 30 min in the dark. After DAPI staining, a confocal microscope (Zeiss, Jena, Germany) was utilized to take photos of EdU cells and the rate of EdU positive cells was quantified with ImageJ software (NIH, Rockville, USA). Cell proliferation rate = EdU positive cell number / total cell number × 100%.

### Limiting dilution analysis

GSC23 or GSC91 cell spheres were trypsinized into individual cells with accutase trypsin (Invitrogen, USA), and plated in 6-well Matrigel pre-coated transfection plates. After 24 h of transfection, GSC cells were counted and cultured in 96-well plates in ten plicate containing 100 µL GSC medium at different densities (100, 50, 20, 10 cells/well). The formation of tumorspheres was determined in each well after 10 days, and GSC frequency was calculated by extreme limiting dilution analysis (http://bioinf.wehi.edu.au/software/elda/).

### Wound healing assay

The wound healing test was performed to prove the effect on cell migration. Cells were seeded in a 6-well plate and allowed to reach a confluence of 90-100% on the second day. A 1.0 mL pipette tip was used to draw a straight line on the surface of the cell to create the wound surface. The separated cells were removed and living cells were maintained with FBS-free DMEM. The starting point was marked with a marking pen on the bottom of the plate. Wound images were taken under a microscope (EVOS, USA) at 0 h, 22 h, or 40 h, and the wound gap area was quantified by ImageJ software.

### Cell invasion and migration assay

In the invasion experiment, Matrigel (DMEM 1:8 dilution, Corning, USA) was pre-coated in a transwell chamber (Corning, USA) and soaked at 37 °C for 2 h before the experiment. 5 × 10^4^ cells in serum-free DMEM were added to the upper wells of the chamber, whereas DMEM with 20%FBS were added to the lower chamber. The chambers were incubated for 24 h in an incubator. Then, the chambers were fixed with 4% PFA and stained with 0.5% crystal violet. The Matrigel and cells on the upper chamber were removed with a cotton swab. The image of invading cells was captured under an optical microscope and analyzed with Image J software. The migration test was performed using the invasion test method, but the Matrigel precoating chamber was not used.

### RNA extraction and quantitative Real-Time PCR assay

Total RNA was isolated from human glioma cells and tissues by TRIZOL reagent (Invitrogen, CA, USA). RNA was quantified with DeNovix DS-11 spectrophotometer (DeNovix, Wilmington, USA), and then reverse transcribed into cDNA by a cDNA synthesis kit (Invitrogen, CA, USA). Quantitative reverse transcription-polymerase chain reaction (qRT-PCR) was performed using SYBR Green (Vazyme, Nanjing, China) in an ABI 7000 thermal cycler. The 2^−ΔΔCT^ method was used to analyze RNA expression. GAPDH was utilized as an internal control for normalization purposes. The primer sequences were synthesized by Sangon Biotechnology (Shanghai, China) and are shown in Table S1.

### Immunoblot assay

RIPA lysis buffer supplemented with protease and phosphatase inhibitors (Beyotime, Shanghai, China) was used to separate total protein from human glioma cells and tissues. The concentration of total protein was determined with a BCA kit (Solarbio, Beijing, China). An equal amount of total protein (30 µg) was electrophoresed on 10%−12% SDS-PAGE, and then electro-transferred to a nitrocellulose filter (NC) membrane (Millipore, MA, USA). Then, the membranes were blocked with 5% non-fat dry milk in TBST at RT for 1 h. After washing with TBST, the membranes were incubated with subsequent primary antibodies at 4 °C overnight. On the second day, the membranes were incubated with HRP-labeled secondary antibodies (Proteintech, Wuhan, China) at RT for 1 h. An enhanced chemiluminescence system (Millipore, MA, USA) was used to measure protein expression value. The relative quantity of proteins was analyzed by Image J software. The antibodies are shown in Table S2.

### Immunofluorescence assay

The cells were cultured in 35 mm glass-bottom dishes with applicable density and transfected with MEOX2 siRNA or CTSS siRNA or Scramble siRNA by Lipofectamine 3000. After 48 h, the cells were washed with pre-cold PBS and fixed with 4% PFA for 30 min. The cells were then incubated with 0.1 % Triton X-100 for 5 min at RT. After washing with PBS, the cells were blocked with 10% BSA at RT for 1 h before being incubated with target primary antibodies overnight at 4 °C. On the second day, the cells were incubated with fluorescent secondary antibodies (Alexa Fluor® 488 or Alexa Fluor® 555) at RT for 1 h in the dark. Finally, the cell nucleus was stained with DAPI, and images of immunofluorescence staining were captured by a confocal microscope. The antibodies are shown in Table S2.

### RNA sequencing analysis

Total RNA was isolated from MEOX2 knockdown SNB19 cells, MEOX2 overexpression U87 cells, and corresponding control SNB19, U87 cells by TRIZOL reagent (Invitrogen, USA). The work related to library construction and sequencing was carried out by Life Gene Biotechnology Co., Ltd. (Shanghai, China). The significance of differentially expressed genes (DEGs) with log_2_(fold change) > 1 and *P* < 0.05 was determined. The raw data of RNA sequencing was uploaded to GEO and the accession number is GSE197758.

### Chromatin immunoprecipitation (ChIP) qPCR

ChIP-qPCR was performed as described previously with minor modification [16]. Briefly, the cells were crosslinked with 1% PFA for 10 min at RT, and then 0.125 M glycine was added to stop the crosslinking reaction. After washing with pre-cold PBS, the cells were centrifuged, lysed with buffer containing Protease Inhibitor Cocktail, and centrifuged again to obtain cell nuclear. The cell nuclear was ultra-sonicated for 6 min with 4 s ultra-sonication at 8 s intervals, added elution buffer containing RNase A, and incubated with Proteinase K at 62 °C for 2 h. The fragmented chromatin extract was incubated with antibodies overnight and then incubated with Protein A/G magnetic beads at 4 °C for 2 h. After thorough washing, elution, and reverse cross-linking, the DNA is purified for qPCR analysis. Production of qPCR reaction was subjected to gel electrophoresis. The primers used for ChIP-qPCR analysis in the promoter are listed in Table S1, and the antibodies are shown in Table S2.

### Luciferase reporter assay

To obtain the constructs of the promoter-luciferase reporter gene, the different promoter regions of the CTSS gene were cloned into the upstream of the luciferase reporter gene of the pGL3.0 vector (GenePharma, Suzhou, China). The cells were plated in 24-well plates in triplicate and allowed to settle for 24 h. Then, the constructs, MEOX2 siRNA and pRL-TK plasmid were co-transfected into SNB19 and U118 cells by Lipofectamine 3000. After transfection of 48 h, the cells were lysed with lysis buffer and performed the dual-luciferase reporter assay by a kit (meilunbio, Dalian, China) according to the manufacturer’s protocol. The sequence of promoter-luciferase reporter constructs in this study is listed in Table S3.

### Immunohistochemistry (IHC) and Hematoxylin–Eosin (H-E) staining

The clinical glioma tissues and brains of xenograft mice were fixed with 4% PFA and embedded in paraffin. Then, 5 µm slices were cut by a microtome (Leica, Germany) and deparaffinized, dehydrated, and incubated in heat-mediated antigen retrieval. Subsequently, the endogenous catalase was eliminated with 3% H_2_O_2_‐methanol, and tissue slices were incubated with indicated primary antibodies at 4 °C overnight. The antibodies are shown in Table S2. After washing with PBS, sections were incubated with biotinylated secondary antibodies at RT for 1 h. They were then incubated with peroxidase solution for 30 min and then the sections were stained with DAB reagent and counterstained with hematoxylin. The images of each section were taken and analyzed under an optical microscope. In HE staining, the paraffin-embedded sections were sequentially deparaffinized, dehydrated, stained by hematoxylin, differentiated by the addition of hydrochloric ethanol, backed to blue with ammonia water, and stained with eosin. Then, the sections were dehydrated with gradient alcohol, cleared with xylene, and sealed with neutral resin. Finally, tissue HE staining photos were acquired under an optical microscope. IHC and HE staining images were quantified by ImageJ software.

### Xenograft tumor assay

All mouse experiments were approved by the Institutional Animal Care and Use Committee of Soochow University. GSC23 cells (3 × 10^5^ in 10 µL PBS) or GSC91 cells (1×10^5^ in 10 µL PBS) or U87 cells (5 × 10^5^ in 10 µL PBS) were injected intracranially into female 6-week-old BALB/c nude mice (SLAC Laboratory Animal Company, Shanghai, China) (5 mice for each group). Mice were observed daily for death or neurological symptoms and then sacrificed if they developed neurological symptoms. The entire brain was then collected, fixed with 4% PFA, embedded in paraffin, and sectioned coronally from anterior to posterior. To measure the size of the tumor, the largest cross-section area of the tumor was selected. The formula for calculating the tumor volume is V = (a × b^2^) / 2, where a is the longest diameter and b is the shortest diameter. a and b are measured with Image J. For survival analysis assays, mice (6 mice for each group) were injected intracranially with the above procedure. The dying mice were sacrificed under deep anesthesia and the remaining mice were sacrificed 90 days after injection of GSC23 cells.

### Bioinformatics analysis

MEOX2 expression value, copy number variation data, and patient survival were downloaded from the TCGA database (https://www.cancer.gov/about-nci/organization/ccg/research/structural-genomics/tcga). Furthermore, MEOX2 expression and survival time data of patients with glioma from the CGGA database and Rembrandt database also were analyzed in this study by the GlioVis website (http://gliovis.bioinfo.cnio.es/). The samples of survival analysis were divided into high and low groups followed MEOX2 expression value. Genes with CNV status from glioma patients were analyzed with Chi-square (χ2) test and circus-plot were drawn with RCircos package. The relationship between MEOX2 expression and MEOX2 CNV status was also analyzed by Chi-square (χ2) test. GSEA was performed by the GSEA website (https://www.gsea-msigdb.org/gsea/index.jsp). Graphic production and statistical analysis were performed by GraphPad Prism 8.0 (GraphPad Software, La Jolla, CA, USA).

### Statistical analysis

Statistical analysis was performed using SPSS 21.0 (SPSS, Chicago, USA) and GraphPad Prism 8.0. Bars and error represent mean ± standard deviation (mean ± SD) of least three independent replicate measurements. The means of normally distributed continuous data between two groups were analyzed by unpaired Student’s *t-*tests. The one-way ANOVA was applied for the comparison of MEOX2 expression levels in clinical different grade glioma. The Chi-square (χ2) test was analyzed the association of MEOX2 expression with the clinicopathological characteristics of glioma. Survival curves were plotted by Kaplan-Meier and compared by log-rank test. Statistical significance was defined as *P* < 0.05.

## Results

### Elevated expression of MEOX2 correlates with poor prognosis in gliomas

**Fig. 1:**
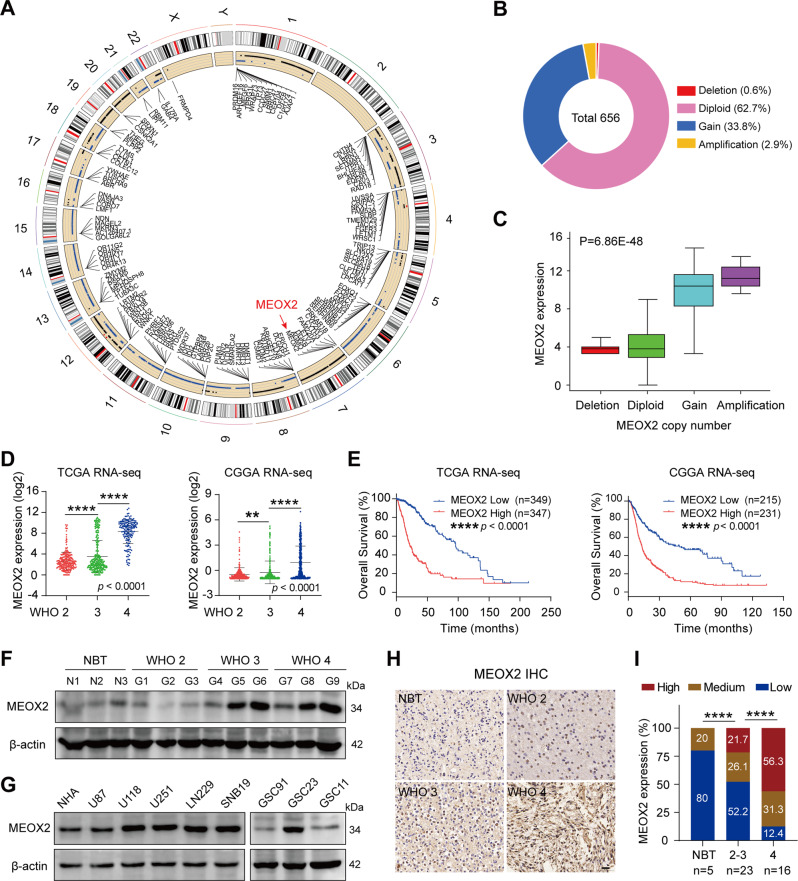
MEOX2 shows overabundance in glioma and is associated with a poor prognosis. **A** Circos plot displayed the genes with a high frequency of CNV change on human chromosomes. The red arrow denotes MEOX2. **B** Different types of MEOX2 genetic alterations in glioma samples from TCGA database (*n* = 656). **C** The correlation of MEOX2 expression with its CNV. **D** The mRNA levels of MEOX2 in gliomas with different WHO grades from TCGA and CGGA databases. **E** Overall survival (OS) curves of patients with glioma from TCGA and CGGA databases stratified by MEOX2 expression. **F** The protein expression of MEOX2 in gliomas with different histological grades was detected by immunoblot. **G** The protein expression of MEOX2 in different glioma cell lines or glioma stem cells and NHA were evaluated by immunoblot. **H** Representative IHC images of MEOX2 protein in gliomas with different histological grades and NBT. Bar = 20 µm. **I** The semi‑quantitative for the IHC results of MEOX2. **p < 0.01, ****p < 0.0001.

Most gliomas are characterized by chromosomal copy number variations (CNV), including gain of chromosome 7 and loss of chromosome 10 [17]. CNV data for glioma patients from TCGA database was analyzed, and genes that are on the various chromosomes with higher rate of CNV were listed in the circos-plot (Fig. 1A). MEOX2, located at chromosome 7pter, attracted our interest. We analyzed different types of MEOX2 genetic alterations in glioma, and found that MEOX2 harbored a large gain or amplification genetically (Fig. 1B). Importantly, MEOX2 copy number gain or amplification positively correlates with its abundant expression (Fig. 1C), suggesting that CNV of MEOX2 results in its expression upregulation and MEOX2 also might be a driver gene for gain of chromosome 7.

To understand the prognostic value of MEOX2 in gliomas, we analyzed MEOX2 expression in public databases. MEOX2 was increased in gliomas with higher WHO grade in TCGA, CGGA, Rembrandt databases (Fig. 1D and Fig. S1A). Glioma and GBM patients with high MEOX2 expression possessed a poorer prognosis (Fig. 1E and Fig. S1B, C). Importantly, univariate analysis showed that besides high MEOX2 expression, MEOX2 CNV status also was the significant factor correlated with survival time. Multivariable Cox regression analysis revealed that high MEOX2 expression correlated with poor patient overall survival, independent of age and MEOX2 CNV status (Table S4). Immunoblotting and RT-PCR analysis results showed that mRNA and protein levels of MEOX2 in fresh glioma tissues were significantly higher than it’s in NBT, especially in Grade3, 4 gliomas (Fig. 1F and Fig. S1D). Additionally, we observed that MEOX2 expression was higher in most glioma cells (U118, U251, SNB19, LN229, GSC23) than NHA (Fig. 1G). Immunohistochemistry (IHC) results also revealed that MEOX2 was increased with increasing WHO grades in glioma, and higher than NBT (Fig. 1H, I). Collectively, the above data indicate that elevated expression of MEOX2 may play an oncogenic role in glioma.

### Depletion of MEOX2 inhibits proliferation, invasion, and migration of glioma cells

**Fig. 2:**
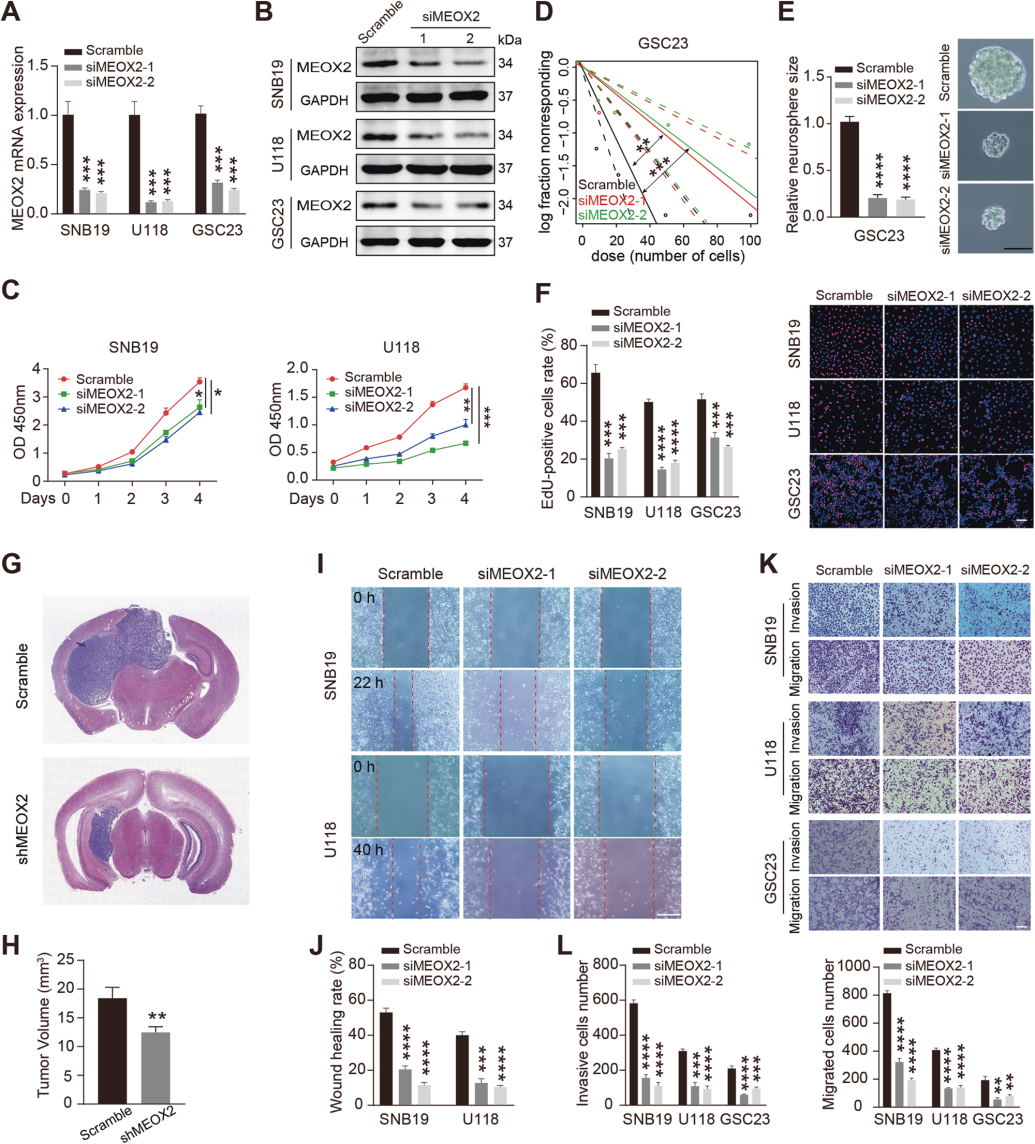
MEOX2 silencing inhibits cell proliferation and motility in glioma. **A**, **B** The mRNA (**A**) and protein (**B**) levels of MEOX2 in SNB19, U118, GSC23 cells transfected with MEOX2 siRNAs or Scramble siRNA. **C** CCK-8 assay was performed to detect cell viability in SNB19 and U118 cells with MEOX2 siRNAs or Scramble siRNA transfection. **D** Limiting dilution assay was performed in GSC23 cells with or without MEOX2 silencing. **E** Representative images of sphere formation capability of GSC23 cells with or without MEOX2 inhibition. Bar = 100 µm. **F** The effect of MEOX2 on cell proliferation in SNB19, U118, GSC23 cells was examined by EdU assay. Bar = 50 µm. **G** shMEOX2 GSC23 cells or control cells were intracranially injected into nude mice, and representative tumor xenografts of HE staining images are shown. **H** The quantitative for the tumor volumes. **I** Wound-healing assay was used to measure wound closure in SNB19 and U118 cells treated with MEOX2 siRNAs or Scramble siRNA. Bar = 400 µm. **J** The quantitative for the wound gap closure. **K** Invasion and migration capabilities were determined by transwell assay in SNB19, U118, GSC23 cells with MEOX2 siRNAs or Scramble siRNA transfection. Bar = 200 µm. **L** The quantitative for the invasive and migrated cells number. *p < 0.05, **p < 0.01, ***p < 0.001, ****p < 0.0001.

To investigate the role of MEOX2 in glioma cells, we constructed MEOX2-silenced cell models by transfected siRNAs into SNB19, U118, GSC23 cells (Fig. 2A, B). CCK-8 and EdU assay verified that silencing MEOX2 significantly attenuated the proliferation of glioma cells (Fig. 2C, F). Limiting dilution assay was performed to examine the self-renewal of glioma stem cells. As shown in Fig. 2D, E, MEOX2-depleted GSC23 cells give rise to fewer and smaller spheres. Cell cycle and apoptosis are accompanied by cell growth. Flow cytometry results showed that MEOX2-knockdowned cell phase distribution was arrested at the G2/M phase (Fig. S2A). However, we observed that silencing MEOX2 did not impact cell apoptosis (Fig. S2B). GSC23 shMEOX2 cells were injected intracerebrally to evaluate the impact of MEOX2 on glioma cells growth in vivo, and the tumor size demonstrated that the growth of MEOX2-depleted cells was suppressed (Fig. 2G, H). Further, we performed wound-healing and transwell assay to examine cell motility, and found that silencing MEOX2 in glioma cells resulted in inhibition of cell migration and invasion (Fig. 2I–L). Together, these results suggest that MEOX2 knockdown significantly inhibited glioma cell proliferation in vitro and in vivo, and also suppressed cell motility.

### Overexpression of MEOX2 facilitates proliferation, invasion, and migration of glioma cells

**Fig. 3:**
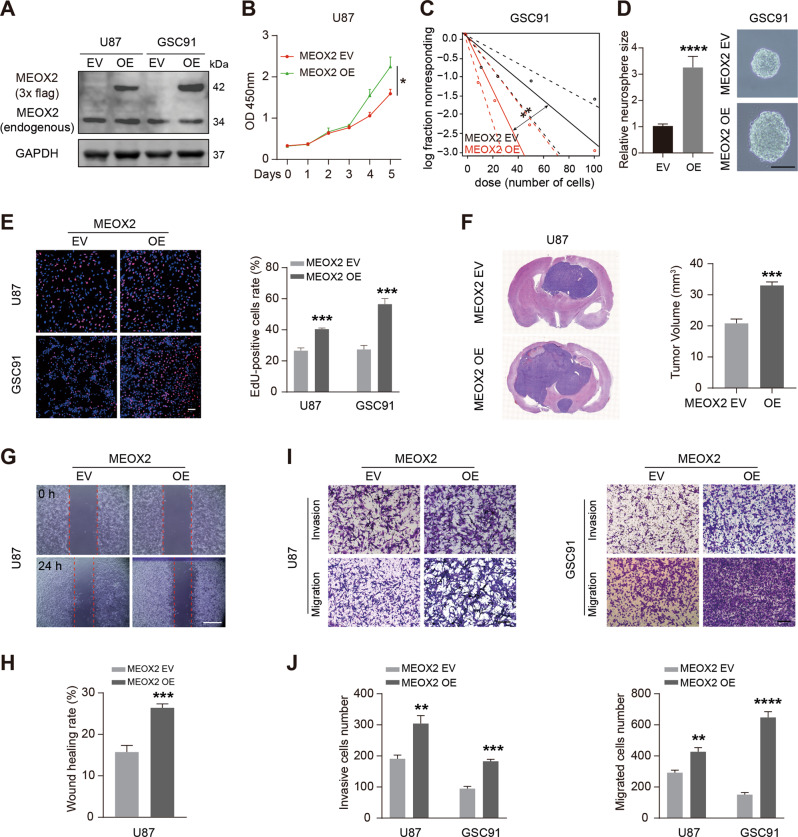
MEOX2 overexpression promotes cell proliferation and motility in glioma. **A** MEOX2 protein expression in U87 and GSC91 cells transfected with MEOX2-Flag or empty vector lentivirus was detected by immunoblot. **B** CCK-8 assay was carried out to test the viability of MEOX2-overexpressed U87 cells or control cells. **C** Limiting dilution assay was performed in GSC91 cells with or without MEOX2 overexpression. **D** Representative images of sphere formation capability of GSC91 cells with or without MEOX2 overexpression. Bar = 100 µm. **E** EdU assay was utilized to measure cell proliferation of MEOX2-overexpressed U87 and GSC91 cells or control cells. Bar = 50 µm. **F** MEOX2-overexpressed U87 cells or control cells were intracranially injected into nude mice, and representative tumor xenografts of HE staining images are shown. **G** Effect of MEOX2 overexpression on U87 cell migration evaluated by wound healing assay. Bar = 400 µm. **H** The quantitative for the wound gap closure. **I** Invasion and migration capabilities were examined by transwell assay in U87 and GSC91 cells with or without MEOX2 overexpression. Bar = 200 µm. **J** The quantitative for the invasive and migrated cells number. *p < 0.05, **p < 0.01, ***p < 0.001, ****p < 0.0001.

We also constructed MEOX2-overexpressed cell models by transfected MEOX2-Flag lentivirus into U87, GSC91 cells (Fig. 3A). CCK-8 and EdU assay results demonstrated that MEOX2 overexpression enhanced U87 cells proliferation (Fig. 3B, E), and limiting dilution assay showed that MEOX2-overexpressed GSC91 cells harbored more and bigger spheres (Fig. 3C, D). Cell cycle distribution showed that MEOX2 overexpression induced fewer cell rates at the G2/M phase (Fig. S2C). HE images revealed that MEOX2-overexpressed glioma cells induced a bigger intracerebral tumor (Fig. 3F and Fig. S2D). Wound-healing and transwell assay verified that MEOX2 overexpression enhanced the invasion and migration abilities of glioma cells (Fig. 3G–J). Collectively, these data indicate that MEOX2 overexpression evidently promoted glioma cell proliferation in vitro and in vivo, as well as contributed to cell motility.

### MEOX2 regulates EMT process, formation of focal adhesion, and F-actin assembly in glioma cells

**Fig. 4:**
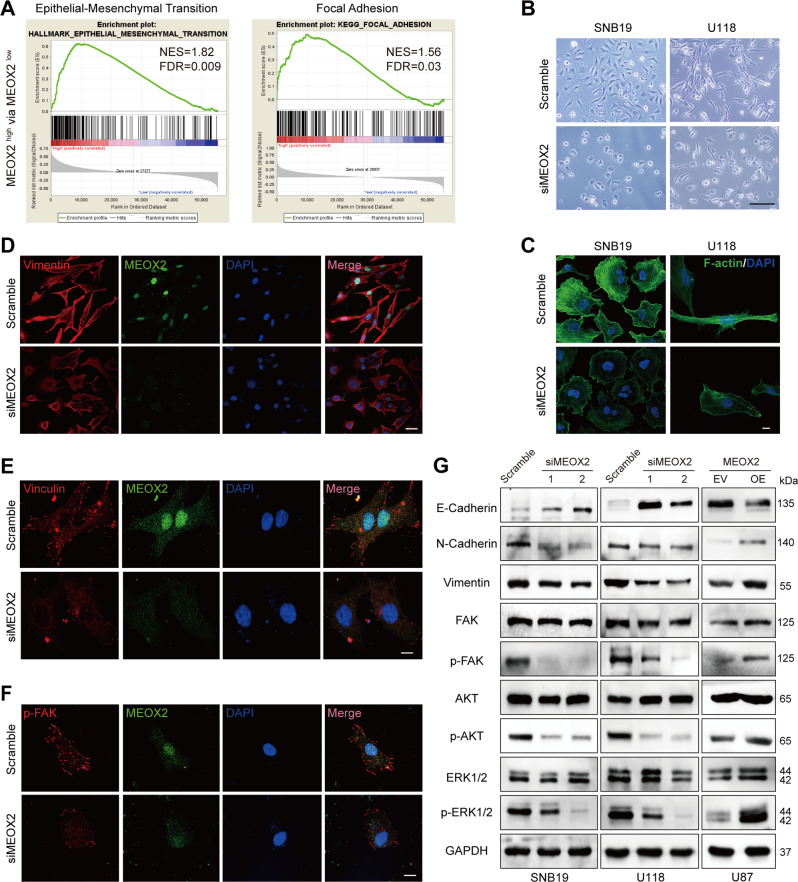
MEOX2 mediates EMT process, formation of focal adhesion, and F-actin assembly in glioma cells. **A** GSEA enrichment terms of EMT and focal adhesion in high MEOX2 expression vs low MEOX2 expression in TCGA gliomas. **B** The morphology of SNB19 and U118 cells treated with MEOX2 or Scramble siRNA was imaged by microscopy. Bar = 200 µm. **C** F-actin immunofluorescence staining of SNB19 and U118 cells with MEOX2 knockdown or control cells were observed by confocal microscopy. Bar = 200 µm. **D**–**F** Vimentin (**D**), Vinculin (**E**), and p-FAK (Y925) (**F**) immunofluorescence staining images of U118 cells with MEOX2 silencing or control cells were captured by confocal microscopy. Bar = 50, 100, 100 µm. **G** The protein expression of E-cadherin, N-cadherin, Vimentin, FAK, p-FAK (Y925), AKT, p-AKT (Ser473), ERK1/2, and p-ERK1/2 (Thr202/Tyr204) were analyzed by immunoblot in SNB19, U118 cells with or without MEOX2 inhibition and U87 cells with or without MEOX2 overexpression.

To get insight into the molecular mechanism underlying MEOX2 mediated glioma malignant phenotype, we analyzed the enriched pathways of genes that positively associated with MEOX2 expression in TCGA, CGGA datasets. GSEA results showed that epithelial-mesenchymal transition (EMT) and focal adhesion were the most significant correlation with MEOX2 expression in glioma (Fig. 4A and Fig. S3A). Interestingly, under a microscope, we observed that the morphology of MEOX2-silenced SNB19 and U118 cells displayed a shortened the length of the pseudopodia (Fig. 4B and Fig. S3B), which represents a typical feature of EMT [18]. We also knock-downed MEOX2 in LN229 cells, and similar cell morphology changes was observed; however, was not observed in NHA cells (Fig. S3C, D). The essential role of actin filament in maintaining the cytoskeleton networks that determine cell shape and motility has been fully documented [19, 20]. Therefore, phalloidin staining measured cytoskeletal actin filament in MEOX2-silenced glioma cells, and the actin filaments displayed a broken and sparse status (Fig. 4C and Fig. S3E). Vimentin, functions as a critical protein involved in cell attachment, migration, signaling, is responsible for maintaining cell shape and stabilizing cytoskeletal interactions, and mediating the EMT process of cancer cells [21]. Immunofluorescence images of vimentin revealed that silencing MEOX2 resulted in significant downregulation of vimentin in glioma cells (Fig. 4D, Fig. S3F and Fig. S4A). Next, vinculin and p-FAK (Y925), molecules of focal adhesion, were also determined using immunofluorescence, and revealed that vinculin and p-FAK (Y925) expression were suppressed in MEOX2 knockdown cells (Fig. 4E, F, Fig. S3G, H and Fig. S4B, C). Besides, immunoblotting results demonstrated that silencing MEOX2 increased E-cadherin protein expression while decreasing the expression of N-cadherin and vimentin, whereas MEOX2 overexpression decreased E-cadherin protein expression while increasing the level of N-cadherin and vimentin. (Fig. 4G, Fig. S3I and Fig. S4D). FAK is a crucial mediator in the focal adhesion pathway since it is required for the creation of nascent adhesions, focal adhesion formation, and F-actin assembly [22–24]. The level of phosphorylated FAK(Y925) was significantly decreased in MEOX2 silenced glioma cells, while increasing in MEOX2 overexpression cells (Fig. 4G, Fig. S3I and Fig. S4D). AKT and ERK1/2 were downstream mediators of FAK pathway, and FAK/AKT/ERK1/2 axis is one of the focal adhesion pathways that are important for cell proliferation and motility [25, 26]. Immunoblot findings revealed that p-AKT (Ser473) and p-ERK1/2(Thr202/Tyr204) proteins expression were obviously decreased in MEOX2 knockdown cells, while were significantly increased in MEOX2 overexpression cells (Fig. 4G, Fig. S3I and Fig. S4D). In summary, these results suggest that MEOX2 may regulate the EMT process, formation of focal adhesion, and F-actin assembly in glioma cells.

### MEOX2 directly targets the regulation of CTSS

**Fig. 5:**
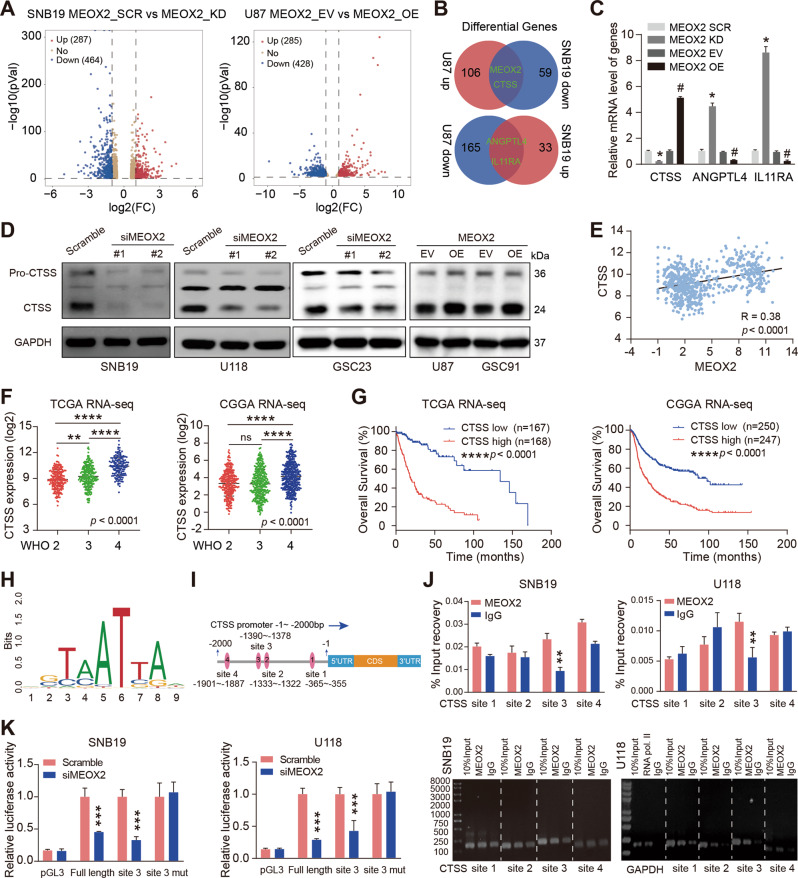
CTSS is a direct transcriptional target of MEOX2. **A** Volcano diagrams display differential genes expression in SNB19 cells with MEOX2 silencing (left), and U87 cells with MEOX2 overexpression (right). **B** Venn diagram depicts the overlap genes between upregulation in U87 cells and downregulation in SNB19 cells, or between upregulation in SNB19 cells and downregulation in U87 cells. **C** RT-PCR analysis was performed to determine the relative mRNA levels of CTSS, ANGPTL4, IL11RA in SNB19 cells with MEOX2 inhibition and U87 cells with MEOX2 overexpression or control cells. **D** Immunoblot of CTSS protein levels in SNB19, U118, GSC23 cells transfected with MEOX2 siRNAs or Scramble siRNA, U87, GSC91 cells which were overexpressed with MEOX2 or EV. **E** The correlation between MEOX2 and CTSS expression in gliomas from TCGA database. **F** The mRNA expression of CTSS in gliomas with different WHO grades from TCGA and CGGA databases. **G** Overall survival (OS) curves of patients with glioma from TCGA and CGGA databases stratified by CTSS expression. **H** The DNA-binding motif of MEOX2. **I** Schematic depicting the detailed information of the potential MEOX2-binding sites on CTSS promoter are shown. **J** ChIP-qPCR analysis of MEOX2 binding on CTSS promoter in SNB19 and U118 cells. **K** Luciferase reporter assays were utilized to detect the transcriptional activity of full length or different clipped forms of CTSS promoter in SNB19 and U118 cells which were transfected with MEOX2 siRNA or Scramble siRNA. ns: no significant, # *p* < 0.05, *p < 0.05, **p < 0.01, ***p < 0.001, ****p < 0.0001.

To identify potential downstream targets of MEOX2 in glioma cells, RNA sequencing was performed. As shown in Fig. 5A, the volcano map (left) displayed that there are 287 upregulated and 464 downregulated in MEOX2 knockdown cells; the volcano map (right) revealed that there are 285 upregulated and 428 downregulated upon MEOX2 overexpression (>2 folds, *p* < 0.05). Further, over-lapping analysis (>4 folds, *p* < 0.01) revealed that there are 3 differential genes (CTSS, ANGPTL4, IL11RA) as candidate downstream genes of MEOX2 (Fig. 5B). RT-PCR analysis validated that the expression of CTSS had the most significant difference in MEOX2 knockdown or overexpression cells (Fig. 5C). Next, immunoblotting verified that silencing MEOX2 significantly suppressed CTSS protein expression, and overexpression of MEOX2 resulted in CTSS upregulation in glioma cells (Fig. 5D). We analyzed the expression data of MEOX2 and CTSS in TCGA dataset, CTSS expression was found to be closely correlated with MEOX2 in glioma (Fig. 5E). Bioinformatics analysis of public databases results found upregulation of CTSS in glioma, especially in WHO 4 glioma, and glioma patients with high CTSS expression possessed poorer prognosis (Fig. 5F, G and Fig. S5A, B). Furthermore, we identified whether MEOX2 directly regulated CTSS expression. As a transcription factor, we predicted four binding sites for MEOX2 on CTSS promoter by JASPAR website (https://jaspar.genereg.net/) (Fig. 5H, I). ChIP-qPCR assay confirmed that MEOX2 is recruited to the site 3 region (-1390 ~ -1378 bp) of CTSS promoter (Fig. 5J). Then, the full-length promoter (-2006 ~ +6 bp) sequence of CTSS, clipped forms of CTSS promoter (site 3, -1515 ~ -1346 bp), and full-length promoter sequence of CTSS with site 3 mutation were cloned into pGL3.0 vector, and luciferase reporter assays demonstrated that silencing MEOX2 inhibited transcriptional activity of the CTSS promoter, but not that of the CTSS promoter with site 3 mutation (Fig. 5K). It suggested that the site 3 region of CTSS promoter may be the only binding region combined with MEOX2. Together, these data confirmed that CTSS acts as a direct target gene of MEOX2 and is positively regulated by MEOX2 in glioma cells.

### Silencing CTSS inhibits proliferation, invasion, and migration of glioma cells

**Fig. 6:**
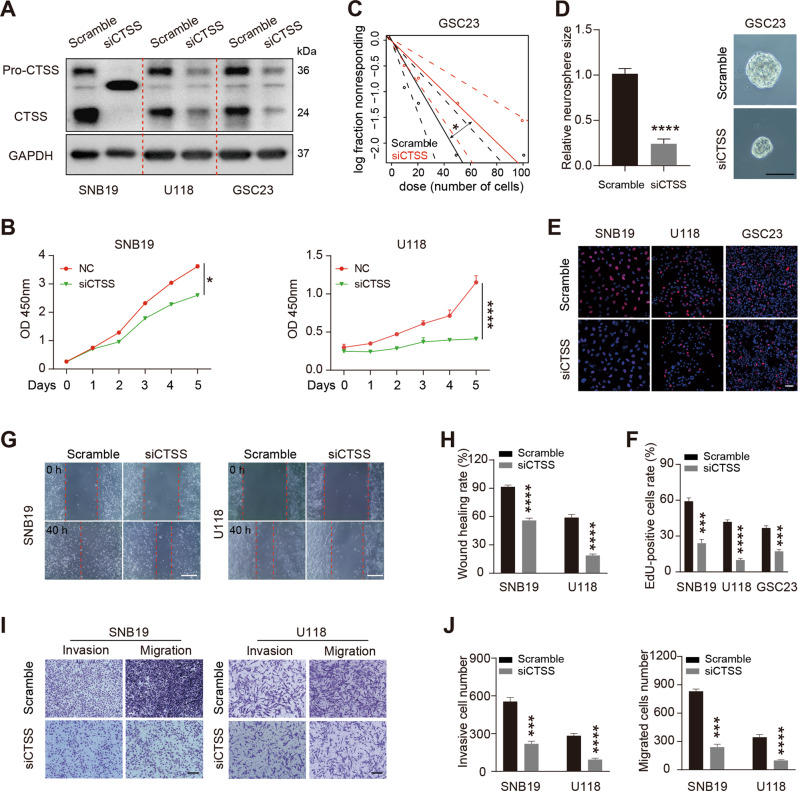
CTSS depletion retards glioma cell proliferation and motility. **A** Western blotting analysis of CTSS expression in CTSS-silenced SNB19, U118, GSC23 cells or control cells. **B** Cell viability was measured in SNB19 and U118 cells with CTSS siRNA or Scramble siRNA transfection. **C** The self-renewal capability of CTSS knockdown GSC23 cells or control cells was determined by limiting dilution assay. **D** Representative images of sphere formation capability of GSC23 cells with or without CTSS inhibition. Bar = 100 µm. **E** Representative EdU fluorescence images of control, siCTSS-treated SNB19, U118, GSC23 cells. Bar = 50 µm. **F** Quantification of the EdU positive cells rate. **G** Wound-healing assay was performed to measure the migration ability of CTSS-knockdown SNB19 and U118 cells or control cells. Bar = 400 µm. **H** Quantification of the wound healing rate. **I** Transwell assay was utilized to assess the invasion and migration capacities in SNB19, U118 cells with CTSS inhibition or control cells. Bar = 100 µm. **J** Quantification of the invasive and migrated cells number. *p < 0.05, ***p < 0.001, ****p < 0.0001.

To identify the role of CTSS in aggressive behavior of glioma cells, we silenced the expression of CTSS in glioma cells by siRNA transfection (Fig. 6A). Then, CCK-8 and EdU assay results showed that silencing CTSS clearly inhibited the proliferation of glioma cells (Fig. 6B, E, F). Moreover, the CTSS-depleted GSC23 cells give rise to fewer and smaller spheres (Fig. 6C, D). Further, we investigated the effect of CTSS on cell motility. Wound-healing and transwell assay confirmed that silencing CTSS evidently inhibited glioma cells invasion and migration (Fig. 6G–J and Fig. S5C, D). Taken together, these data suggest that silencing CTSS inhibits cell proliferation, invasion, and migration of glioma.

### CTSS mediates EMT process, formation of focal adhesion, and F-actin assembly in glioma cells

**Fig. 7:**
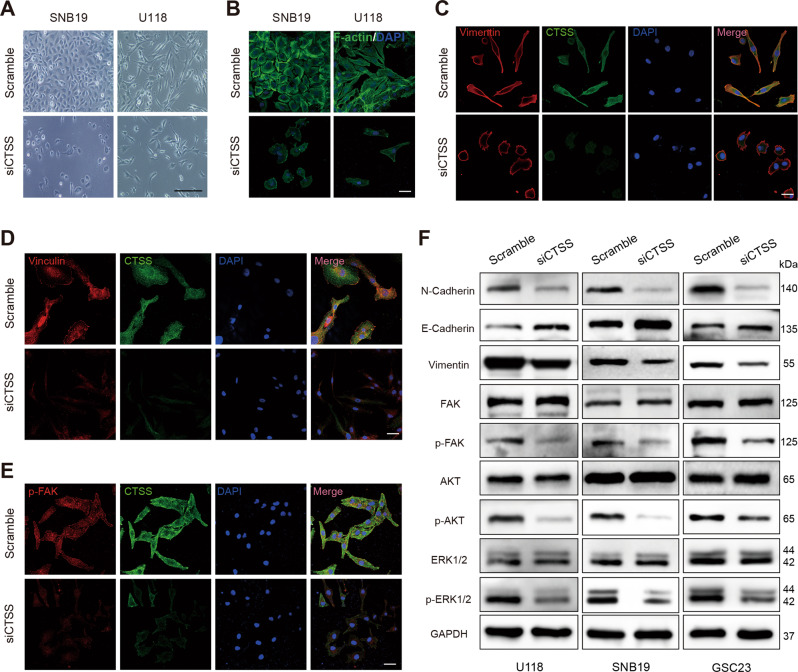
CTSS regulates EMT process, formation of focal adhesion, and F-actin assembly in glioma cells. **A** The morphology of SNB19 and U118 cells with or without CTSS inhibition was imaged by microscope. Bar = 200 µm. **B** F-actin immunofluorescence images of SNB19 and U118 cells with CTSS siRNA or Scramble siRNA transfection were captured by a confocal microscope. Bar = 40 µm. **C**–**E** Vimentin (**C**), Vinculin (**D**), and p-FAK (Y925) (**E**) immunofluorescence staining of U118 cells with CTSS knockdown or control cells were observed by a confocal microscope. Bar = 40 µm. **F** The protein expression of E-cadherin, N-cadherin, Vimentin, FAK, p-FAK (Y925), AKT, p-AKT (Ser473), ERK1/2, and p-ERK1/2 (Thr202/Tyr204) were analyzed by immunoblot in SNB19, U118, GSC23 cells transfected with CTSS or Scramble siRNA.

The above evidence confirmed that MEOX2 could regulate the EMT process and focal adhesion in glioma cells; moreover, CTSS acts as one downstream mediator of MEOX2. We investigated the role of CTSS on the EMT process, focal adhesion and F-actin assembly in glioma cells. First, the morphology of CTSS-silenced cells was observed under a microscope, and also displayed a shortened the length of the pseudopodia (Fig. 7A and Fig. S5E). Phalloidin staining images showed that actin filaments of CTSS-silenced cells were broken and reduced (Fig. 7B and Fig. S5F). Further, immunofluorescence staining revealed that vimentin, vinculin, p-FAK (Y925) expression were obviously reduced in CTSS knockdown cells (Fig. 7C–E, Fig. S5G-I and Fig. S6A-C). Additionally, the immunoblotting analysis demonstrated that E-cadherin protein expression was upregulated, while N-cadherin, vimentin, p-FAK (Y925) was downregulated in CTSS-silenced cells (Fig. 7F and Fig. S5J). Moreover, p-AKT (Ser473) and p-ERK1/2(Thr202/Tyr204) were decreased in CTSS knockdown cells (Fig. 7F and Fig. S5J), and it meant that AKT/ERK1/2 signaling axis was inactivated in CTSS-depleted glioma cells. Collectively, these data demonstrated that what the role of CTSS in aggressive behavior and its molecular mechanism in glioma cells is similar to MEOX2, also indicated that MEOX2 contributed to the malignant behavior of glioma by CTSS activation.

### MEOX2 induces glioma malignant phenotype via CTSS in vitro and in vivo

**Fig. 8:**
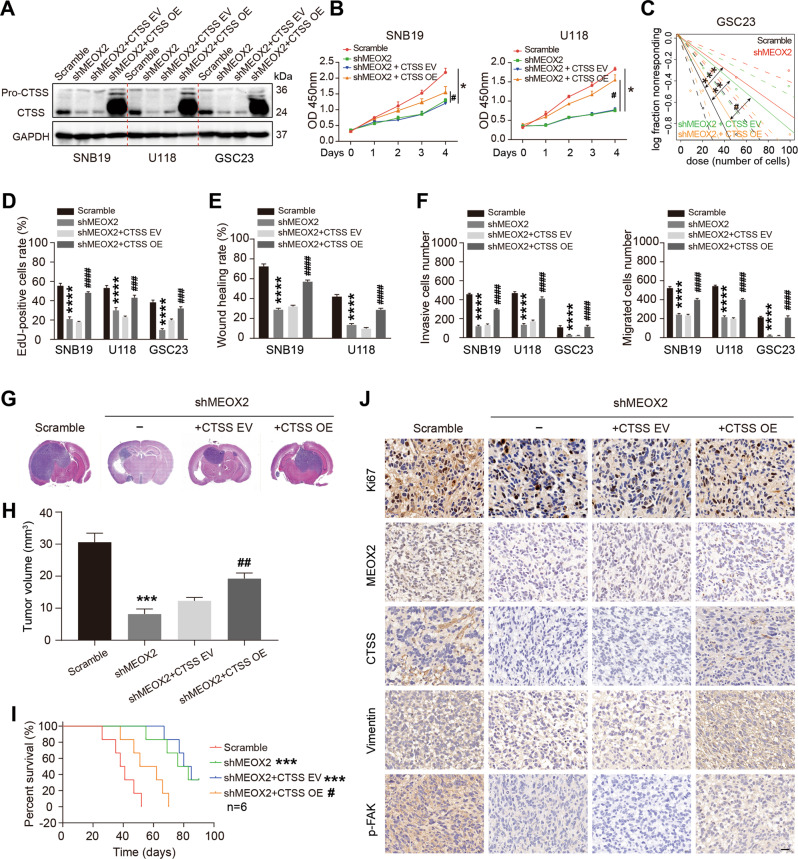
CTSS re-expression partially abrogates cell proliferation and motility inhibition caused by MEOX2 silencing in glioma. **A** Immunoblot analysis of CTSS expression in SNB19, U118, GSC23 cells treated with Scramble shRNA, shMEOX2, shMEOX2 + CTSS EV and shMEOX2 + CTSS OE. **B** Cell viability of SNB19 and U118 cells infected with Scramble shRNA, shMEOX2, shMEOX2 + CTSS EV and shMEOX2 + CTSS OE was evaluated by CCK-8 assay. **C** The self-renewal capability of GSC23 cells treated with Scramble shRNA, shMEOX2, shMEOX2 + CTSS EV and shMEOX2 + CTSS OE was assessed by limiting dilution assay. **D** Cell proliferation of SNB19, U118, GSC23 cells infected with Scramble shRNA, shMEOX2, shMEOX2 + CTSS EV and shMEOX2 + CTSS OE was determined by EdU assay. **E** Cell migration ability of SNB19 and U118 cells infected with Scramble shRNA, shMEOX2, shMEOX2 + CTSS EV and shMEOX2 + CTSS OE was evaluated by wound healing assay. **F** Cell invasion and migration capacities of SNB19, U118 and GSC23 cells treated with Scramble shRNA, shMEOX2, shMEOX2 + CTSS EV and shMEOX2 + CTSS OE was detected by transwell assays. **G** Control, shMEOX2, shMEOX2 + CTSS EV and shMEOX2 + CTSS OE-infected GSC23 cells were intracranially injected into nude mice, and representative HE images of xenograft tumors are shown. **H** Quantification of the tumor volumes. **I** Kaplan-Meier survival curve shows the survival times of GSC23 scramble, GSC23 shMEOX2, GSC23 shMEOX2 + CTSS EV and GSC23 shMEOX2 + CTSS OE xenograft mice. **J** IHC staining representative images show Ki67, MEOX2, CTSS, Vimentin and p-FAK expression in GSC23 scramble, GSC23 shMEOX2, GSC23 shMEOX2 + CTSS EV and GSC23 shMEOX2 + CTSS OE xenograft mice. Bar = 20 µm. * shMEOX2 treatment group vs control group, *p < 0.05, **p < 0.01, ***p < 0.001, ****p < 0.0001. # shMEOX2 + CTSS OE treatment group vs shMEOX2 + CTSS EV group, ^#^*p* < 0.05, ^##^*p* < 0.01, ^###^*p* < 0.001, ^####^*p* < 0.0001.

To confirm the role of CTSS in MEOX2 inducing glioma malignant phenotype, we constructed stable cell lines by transfected CTSS overexpression lentiviruses into shMEOX2 cells for the rescue assay (Fig. 8A). CCK-8 and EdU assay showed that rescue of CTSS partially restored the viability of MEOX2-silenced glioma cells (Fig. 8B, D and Fig. S7B), and limiting dilution assay revealed that rescue of CTSS also partially restored the self-renewal capacity of MEOX2-depleted GSC23 cells (Fig. 8C and Fig. S7A). Moreover, MEOX2-depleted induced inhibition of glioma cells migration and invasion were partially restored by re-expression of CTSS (Fig. 8E, F and Fig. S7C, D). Further, we investigated the effects of MEOX2-CTSS axis in glioma tumorigenesis in vivo. The results of GSC23 cells xenografting verified that silencing MEOX2 significantly decreased transplant tumor size, whereas the CTSS re-expression partially neutralized the above effect of MEOX2 knockdown (Fig. 8G, H). KM survival analysis demonstrated that the survival time of xenograft mice was noticeably prolonged after silencing MEOX2, while the CTSS rescue xenograft mice had a similar survival time to control mice (Fig. 8I). IHC staining was performed to detect the expression of related molecules in xenograft mice brain sections. Consistent with the results of in vitro studies, IHC staining showed a lower expression of MEOX2, Ki67, CTSS, vimentin, p-FAK in MEOX2-silenced tumors (Fig. 8J and Fig. S7E). In the CTSS rescue GSC23 tumors, we observed partially upregulation of Ki67, vimentin, p-FAK, and significantly upregulation of CTSS compared to MEOX2-silenced tumors; however, the expression of MEOX2 had no differential (Fig. 8J and Fig. S7E). These data further confirm that MEOX2 plays a role in promoting the malignant progression of glioma by regulating the expression of CTSS in vitro and in vivo.

## Discussion

Previous studies documented that MEOX2 existed a dual role in human cancers [9–12], however, was determined an overexpression status in glioma [13, 14]. In our study, we confirmed that glioma had gain and amplification on the MEOX2 locus, which may upregulate MEOX2 expression. Copy number variations (CNVs) have emerged as an important tool in diagnostics for brain tumor and are already leveraged to render a clear diagnosis [27]. We analyzed genes with CNVs based on the TCGA database and focused on genes located on chromosome 7, which is frequency gained in glioma patients. Interestingly, MEOX2, which is located at chromosome 7pter, harbored a large of gain or amplification genetically. Further, MEOX2 copy number gain or amplification positively correlates with its abundant expression. Consistent with the report of Tachon et al., amplification of MEOX2 is strongly associated with chr7 gain and indicates that MEOX2 also might be a driver gene for the gain of chr7 [13]. Our results demonstrated that MEOX2 was upregulated in glioma, particularly in GBM, and it is significantly positively correlated with the prognosis of glioma patients; suggesting that MEOX2 has the potential to be prognostic markers for glioma.

It is well known that homeotic genes, HOXA-Ds, which encode conserved homeodomain transcription factors, have crucial roles in the development of human cancers [28, 29]. However, MEOX2, which is a nonclassical HOX gene, has seldom been reported in human cancers. Bioinformatics analysis of available public databases suggested that MEOX2 is overexpressed in gliomas and correlates with the poor prognosis of patients. Then, both loss-of-function and gain-of-function studies were performed in glioma cell lines and glioma stem cells, confirmed that MEOX2 facilitated cell proliferation of glioma in vitro and in vivo. Further mechanistically indicated that MEOX2 expression was proven to affect AKT and ERK1/2 activation, which mainly mediates the survival and proliferation of cells [30]. Of note, MEOX2-mediated AKT/ERK pathway has been implicated in cell proliferation [31]. Further study found that depletion of MEOX2 inhibited glioma cell proliferation by arresting cell cycle at G2/M phase, however, not that of cell apoptosis.

One of the important findings of this study is that silencing MEOX2 in glioma cells resulted in inhibition of cell motility. A previous study expounded that silencing MEOX2 could weaken the capacity of invasion and migration in NSCLC cells [9]. GSEA analysis indicated that MEOX2 may involve in the process of EMT and focal adhesion in glioma cells, which are the important mechanisms that participate in glioma invasion and migration [32]. Here, we demonstrated that E-cadherin was significantly augmented while N-cadherin, vimentin, and p-FAK/FAK ratio was reduced in glioma cells with MEOX2 knockdown, and the expression of these molecules showed the opposite trend in MEOX2 overexpression glioma cells. Moreover, we noticed that inhibition of MEOX2 impaired the expression of vimentin, vinculin, and p-FAK, indicating a more epithelial phenotype and non-maturation of focal adhesion under MEOX2 inhibition. Interestingly, typical cell morphology changes of EMT were observed in glioma cells with MEOX2 knockdown, however, this was not observed in NHA cells. Furthermore, inhibition of MEOX2 severely impaired F-actin polymerization, which is an essential process for actin assembly and cytoskeleton construction, and also is fundamental for cancer cell motility [24, 33]. Therefore, our findings suggested that MEOX2 was a crucial effector molecule that induced EMT, formation of focal adhesion, and F-actin polymerization to mediate cell motility in glioma.

MEOX2 is a transcription factor that binds to the promoter of target genes to regulate gene transcription. In our study, Cathepsin S (CTSS) was identified as a major downstream target gene of MEOX2 in glioma by RNA-sequencing, although, other target genes may be also regulated via MEOX2. CTSS, which is a member of the cathepsin family of cysteine proteases, has been implicated in an important role in cancer development [34–36], was highly expressed in human cancer. In glioma, CTSS was overexpressed and identified as an independent prognostic factor [37, 38]. Consistently, we analyzed CTSS expression and survival data of glioma patients from the public databases, and demonstrated that CTSS was upregulated and correlated with the poor prognosis. Further, we found that CTSS, which is transcriptionally regulated by MEOX2, contributed to cell proliferation and motility in glioma. The previous study has suggested that CTSS could mediate the process of invasion and migration in glioma [39], however, no reports have clearly confirmed the proliferation effect of CTSS on glioma cells. In our study, depletion of CTSS in glioma cells or glioma stem cells suppressed cell proliferation or self-renewal, indicating CTSS also was a growth regulator in glioma. Importantly, CTSS had a more pronounced effect on regulating cell motility in glioma. The mechanism, inhibition of CTSS induced FAK/AKT/ERK pathway inactivation, blocked the process of EMT and formation of focal adhesion, and reduced actin filaments assembly. These results are consistent with the effects of MEOX2 on glioma, and CTSS re-expression in MEOX2 depleted cells could partially restore the capacities of cell proliferation and motility in vitro and in vivo, suggesting that MEOX2-CTSS axis facilitated the malignant progression of glioma by above molecular mechanisms. CTSS is a lysosomal cysteine protease located at the lysosome and cytoplasm; however, it can be secreted into the tumor microenvironment to induce tumor invasion, angiogenesis, and metastasis [40]. Importantly, CTSS also can promote the degradation of damaged or unwanted proteins in the endo-lysosomal pathway or ubiquitin-proteasome system [35, 41]. In our study, we confirmed that MEOX2-CTSS axis regulated glioma cell growth and motility through intracellular mechanisms. Whether CTSS mediated glioma malignant phenotype by extracellular mechanism needs more profound exploration. In addition, inhibition of MEOX2 impaired cell growth and motility in glioma, while CTSS expression rescue did not entirely restore behavior, implying the presence of other cellular or molecular mechanism.

In conclusion, in the present study, we demonstrated that transcription factor MEOX2 could serve as a poor prognostic indicator in glioma, and MEOX2-CTSS axis contributes to the malignant progression of glioma by regulation of EMT and focal adhesion. Therefore, MEOX2-CTSS axis has the potential to be prognostic markers and therapeutic targets for glioma. Nevertheless, several limitations should be acknowledged. MEOX2 shared the same domain (homeodomain) with homeobox genes, it was not complete to target MEOX2 and preserve the homeodomain to figure out how MEOX2 works. Therefore, validation of the mechanism of the MEOX2 isoform in glioma warrants further investigation. PI3K/AKT, Wnt/β-catenin, Hippo pathways are closely related with EMT and focal adhesion in glioma, thus the mechanism of MEOX2 in glioma might be more complicated than this study presents [24, 42, 43]. Thus, we will further explore the roles and molecular mechanisms of MEOX2 in glioma, and provide insight into novel therapeutic strategies for glioma.

## Supplementary information

Supplementary Materials

aj-checklist

## Data Availability

All data generated or analyzed during this study are included in the article and its supplementary files, and available from the corresponding author on reasonable request.
