# Supplementary material for: MEOX2-mediated regulation of Cathepsin S promotes cell proliferation and motility in glioma
Source: Cell Death Dis. 2022 Apr 18;13(4):360. doi: 10.1038/s41419-022-04845-2 (PMC9016080; doi:10.1038/s41419-022-04845-2)

**Supplementary Materials**

*for*

**MEOX2-mediated regulation of Cathepsin S promotes cell proliferation and motility in glioma**

Ji Wang^1,2^, Yanming Chen^1,2^, Qing Wang^1,2^, Hui Xu^1,2^, Chunwang Wu^1,2^, Qianqian Jiang^1,2^, Guoqing Wu^1,2^, Honglong Zhou^3^, Zongyu Xiao^4^, Ying Chen^1,2^, Tan Zhang^1,2^ and Qing Lan^1,2^

^1^Department of Neurosurgery, The Second Affiliated Hospital of Soochow University, Suzhou 215004, China

^2^Jiangsu Key Laboratory of Neuropsychiatric Disease, Institute of Neuroscience, Soochow University, Suzhou 215123, China

^3^Department of Neurosurgery, The Second Affiliated Hospital of Nanchang University, Nanchang 330006, China

^4^Department of Neurosurgery, Dushu Lake Hospital, Soochow University, Suzhou 215124, China

The supplementary materials include materials and methods, 4 supplementary tables and 7 supplementary figures.

**Materials and methods**

**Cell cycle analysis**

Cell cycle assay was performed to analyze cell cycle phase distribution with a kit (Beyotime, Shanghai, China). The cells were harvested and fixed with pre-cold 70% ethanol at 4 °C overnight. Then, the cells were incubated in a 500 µL staining buffer containing 25 µL Propidium Iodide (PI) and 10 µL RNase A at RT for 30 min in the dark. The stained cells were then immediately analyzed with a CytExpert flow cytometer (Beckman Coulter, USA).

**Cell apoptosis analysis**

The cells were seeded in 6-well plates and transfected with MEOX2 siRNA or Scramble siRNA by Lipofectamine 3000 for 48 h. Then, these cells were collected and washed with PBS and incubated with 210 µL binding buffer containing 5 µL annexin V-FITC and 10 µL PI in the dark for 20 min at RT. Finally, the CytExpert flow cytometer was used to analyze the apoptosis status.

**Table S1. List of primers used in this study.**

| **Primers** | **Sequence (5'-3')** |
| --- | --- |
| **Primers for qRT-PCR** |  |
| h-MEOX2 | Forward: GCGATACGAGATAGCAGTGAATC |
|  | Reverse: GCTGTCCACCCTTTACCCTC |
| h-CTSS | Forward: TGACAACGGCTTTCCAGTACA |
|  | Reverse: GGCAGCACGATATTTTGAGTCAT |
| h-ANGPTL4 | Forward: GGCTCAGTGGACTTCAACCG |
|  | Reverse: CCGTGATGCTATGCACCTTCT |
| h-IL11RA | Forward: TCAAGTTCCGTTTGCAGTACC |
|  | Reverse: GCACTGACTCGTACAGCATGG |
| h-GAPDH | Forward: GGGAGCCAAAAGGGTCAT |
|  | Reverse: GAGTCCTTCCACGATACCAA |
| **Primers for Chip-qPCR** |  |
| h-CTSS (promoter site 1) | Forward: GTGACAGAATGAAACCCTGTCTC |
|  | Reverse: AGGCAAGTCATCTAGGCCAC |
| h-CTSS (promoter site 2) | Forward: AGGACAAGTGGAATTTCATAGGCT |
|  | Reverse: CCGGCCAAGGTGTATGTGTT |
| h-CTSS (promoter site 3) | Forward: ATAGTTCGTCTTGCTCCCACC |
|  | Reverse: CTCAGCCATTCCTTCGTTCATC |
| h-CTSS (promoter site 4) | Forward: TCAGCTGGGACTACAAGCATG |
|  | Reverse: GAGGCCAGGAGTTCAAGACC |
| h-GAPDH | Forward: GGTTTTACGGGCGCACGT |
|  | Reverse: GCTGACTGTCGAACAGGAGG |

**Table S2. List of antibodies used in this study.**

| **Antigen** | **Cat. No** | **Company** | **Dilution** |
| --- | --- | --- | --- |
| **Antibodies for WB** |  |  |  |
| MEOX2 | ab124876 | Abcam | 1:2000 |
| CTSS | 25084 | CST | 1:1000 |
| Vimentin | ab92547 | Abcam | 1:5000 |
| N-Cadherin | ab76011 | Abcam | 1:1000 |
| E-Cadherin | ab40772 | Abcam | 1:2000 |
| AKT | 9272 | CST | 1:1000 |
| p-AKT(Thr308) | 13038 | CST | 1:1000 |
| ERK1/2 | 4695 | CST | 1:1000 |
| p-ERK1/2(Thr202/Tyr204) | 4370 | CST | 1:1000 |
| FAK | ab40794 | Abcam | 1:2000 |
| p-FAK(Tyr925) | 3284 | CST | 1:1000 |
| Flag | 2368 | CST | 1:1000 |
| GAPDH | 60004-1-Ig | Proteintech | 1:5000 |
| β-actin | 66009-1-Ig | Proteintech | 1:5000 |
| **Antibodies for ChIP** |  |  |  |
| MEOX2 | sc-376748 | Santa Cruz | 1:10 |
| IgG | 10400C | Thermo Fisher | 1:100 |
| **Antibodies for IHC** |  |  |  |
| MEOX2 | AP18398C | abcepta | 1:500 |
| Ki67 | ab15580 | Abcam | 1:200 |
| CTSS | sc-271619 | Santa Cruz | 1:50 |
| Vimentin | ab92547 | Abcam | 1:1000 |
| p-FAK(Tyr925) | 3284 | CST | 1:25 |
| **Antibodies for IF** |  |  |  |
| MEOX2 | sc-376748 | Santa Cruz | 1:100 |
| CTSS | sc-271619 | Santa Cruz | 1:100 |
| Vimentin | ab92547 | Abcam | 1:1000 |
| p-FAK(Tyr925) | 3284 | CST | 1:200 |
| Vinculin | ab129002 | Abcam | 1:500 |

**Table S3. List of the sequence of promoter-luciferase reporter constructs in this study.**

**Site 3 of CTSS promoter (-1515~ -1346 bp)**

AAAGATGATAGTTCGTCTTGCTCCCACCCCACCCAGGTAATCATTCTCAATTGGATATATTTGAGTGTATCCTTTTTTCCATGAATATACTAACACATACACACACACACACACACACACACGCATATTACTAAACTATACATATAGTATATATTAATAAAGACATTTT

**CTSS full length promoter (-2006 ~ 6 bp)**

GGTACCTACAAAAAACAAAAAACAAAATCAGCCAGGCATGGTGGTGTGCACCTGTAGTCCCAGCCACTCCAGAGGCTGAGGCGGGAGGATCACTTGAGCCCAGGAGGTCAAGGCTGCAGTGAGCCATGATTGCATCACTGCACTCCAGCCTGGGTGACAGAATGAAACCCTGTCTCCAAAAGAAAAAAGCAAAAAACTCCAACAGAGTTTTTCAGGGAACTTGATAAACTGATTCAAAAAATTCATATAAAGAAGAGTTAACAAGTACAAAATACAAAGACAATGTTTAAAAAGAAGAACAAAGATAGGGTGGCCTAGATGACTTGCCTTACCATACGTGGAGACATATTACAAAGCTGTTTTAATTAAAACTGTGTGATACTGAAGTAGCAAAAGATAAAAAGAACAGCTTCATGAGCTCAGAAACTGACCTATCTATGTATGAGAATTTGGTGTACAATAGAGGTAGAATCAAAATTGGAAAACAAAGAAAATAAGCTCCCTACGTCATACCATACACAAAACTAAATTCCAGGTGAATTAAACACCTAAAAGCAGTAGCCATAGAAAAGATAAATAAAACACTCTCTGAAAGTATAAAAATAAGATGAGTGTATCTTTATTACATTTGGATGAGGAAAATTTTCTTATACAAAAACAGTATAGATTGATTATACGGAAAAGCTGAATAAAATGTTCTACCTCAACATAAAAAAATTTCATATAGAAAAACTCACTATAAACAAGACTAAAGGACAAGTGGAATTTCATAGGCTTATGCAAAGGAAGAAAAAATAAAATAAAAGACAAGTGGAAGACTAAGGGAGGATGTTTTTAAGGGTATATAACTGTCAAAGATTAGAAGCCAGAATATATTTTTAAAACACATACACCTTGGCCGGGCACGGTGGCTCACACCTGTAATCCCAGCACTTTGGGAGGCTGAGGCGGGTGGATCACGAGGTGAAGAGTTCAAGACCAGCCTGGCCAAGATGGTGAAACCCTGTCTCTACTAAAAATACAAAAATTAGCTGGACATGCTGGCAGGTGCCTGTAATCCCAGCTACTTGGGAGTCTGAGGCAGAGAATCGCTTGAACCTGGGAGGTGGATGTTGCAGTGAGCCAAGATCGCACCAATGCACTCCAGCATGGGAGACAAAGCACGACTCCGTCTCGGAAAACACACAACACACACACACACACACACCTCCCACAAATCACTAAGAAAATGCAACAATACAATAGAAAAATGAGCAAAAAAGATGAACAGGAAATTCACAGAAAATTCAAACGGTCAATAAATGTGTGAAAAGATCCTCAACCTCATTAGTAATTAGGAAAATGCAAATTTAAAAGATGATAGTTCGTCTTGCTCCCACCCCACCCAGGTAATCATTCTCAATTGGATATATTTGAGTGTATCCTTTTTTCCATGAATATACTAACACATACACACACACACACACACACACACGCATATTACTAAACTATACATATAGTATATATTAATAAAGACATTTTGAGTTTGATGAACGAAGGAATGGCTGAGTGAGAACCAGACAATATCAGATCATGAATGAGTTGTGTTAAAAGCAGTAAGACAGGTTTTCCTAGGAAATCATACAAGGAGCTGGGATTTGGGGAGCTTTATCTAAATCTCTTTATAACTAAATGTTTTCCATGTAAAAGTTGGTGTTTTTAAAATTCAATTTAATTTTTTTTTTGAGATAGGGTCTTGATATGTCATCCAGGCTGAAACACAGTGGCATAATCATGGCTCACTGCAGCCTTGTCCTCCTAGACTCAAGCAATTCTCCCACCTCACCCTCCCCCTCAGCTGGGACTACAAGCATGCACCACCATGCCCAGCTAATTAAAAAAAATTTTTTTGCAGAGACAGGGTCTCACTATATTGCCTAGGCTGGTCTTGAACTCCTGGCCTCAAGTGATCCTCCCGCCTCAGCCTCCCAAAGTGCTGGGATTATAGGCATGAGCCACTGGGCTCACTCGAG

**CTSS full length promoter with mutation of site 3 (-2006 ~ 6 bp)**

GGTACCTACAAAAAACAAAAAACAAAATCAGCCAGGCATGGTGGTGTGCACCTGTAGTCCCAGCCACTCCAGAGGCTGAGGCGGGAGGATCACTTGAGCCCAGGAGGTCAAGGCTGCAGTGAGCCATGATTGCATCACTGCACTCCAGCCTGGGTGACAGAATGAAACCCTGTCTCCAAAAGAAAAAAGCAAAAAACTCCAACAGAGTTTTTCAGGGAACTTGATAAACTGATTCAAAAAATTCATATAAAGAAGAGTTAACAAGTACAAAATACAAAGACAATGTTTAAAAAGAAGAACAAAGATAGGGTGGCCTAGATGACTTGCCTTACCATACGTGGAGACATATTACAAAGCTGTTTTAATTAAAACTGTGTGATACTGAAGTAGCAAAAGATAAAAAGAACAGCTTCATGAGCTCAGAAACTGACCTATCTATGTATGAGAATTTGGTGTACAATAGAGGTAGAATCAAAATTGGAAAACAAAGAAAATAAGCTCCCTACGTCATACCATACACAAAACTAAATTCCAGGTGAATTAAACACCTAAAAGCAGTAGCCATAGAAAAGATAAATAAAACACTCTCTGAAAGTATAAAAATAAGATGAGTGTATCTTTATTACATTTGGATGAGGAAAATTTTCTTATACAAAAACAGTATAGATTGATTATACGGAAAAGCTGAATAAAATGTTCTACCTCAACATAAAAAAATTTCATATAGAAAAACTCACTATAAACAAGACTAAAGGACAAGTGGAATTTCATAGGCTTATGCAAAGGAAGAAAAAATAAAATAAAAGACAAGTGGAAGACTAAGGGAGGATGTTTTTAAGGGTATATAACTGTCAAAGATTAGAAGCCAGAATATATTTTTAAAACACATACACCTTGGCCGGGCACGGTGGCTCACACCTGTAATCCCAGCACTTTGGGAGGCTGAGGCGGGTGGATCACGAGGTGAAGAGTTCAAGACCAGCCTGGCCAAGATGGTGAAACCCTGTCTCTACTAAAAATACAAAAATTAGCTGGACATGCTGGCAGGTGCCTGTAATCCCAGCTACTTGGGAGTCTGAGGCAGAGAATCGCTTGAACCTGGGAGGTGGATGTTGCAGTGAGCCAAGATCGCACCAATGCACTCCAGCATGGGAGACAAAGCACGACTCCGTCTCGGAAAACACACAACACACACACACACACACACCTCCCACAAATCACTAAGAAAATGCAACAATACAATAGAAAAATGAGCAAAAAAGATGAACAGGAAATTCACAGAAAATTCAAACGGTCAATAAATGTGTGAAAAGATCCTCAACCTCATTAGTAATTAGGAAAATGCAAATTTAAAAGATGATAGTTCGTCTTGCTCCCACCCCACTTGAACGGCTGCTCTCAATTGGATATATTTGAGTGTATCCTTTTTTCCATGAATATACTAACACATACACACACACACACACACACACACGCATATTACTAAACTATACATATAGTATATATTAATAAAGACATTTTGAGTTTGATGAACGAAGGAATGGCTGAGTGAGAACCAGACAATATCAGATCATGAATGAGTTGTGTTAAAAGCAGTAAGACAGGTTTTCCTAGGAAATCATACAAGGAGCTGGGATTTGGGGAGCTTTATCTAAATCTCTTTATAACTAAATGTTTTCCATGTAAAAGTTGGTGTTTTTAAAATTCAATTTAATTTTTTTTTTGAGATAGGGTCTTGATATGTCATCCAGGCTGAAACACAGTGGCATAATCATGGCTCACTGCAGCCTTGTCCTCCTAGACTCAAGCAATTCTCCCACCTCACCCTCCCCCTCAGCTGGGACTACAAGCATGCACCACCATGCCCAGCTAATTAAAAAAAATTTTTTTGCAGAGACAGGGTCTCACTATATTGCCTAGGCTGGTCTTGAACTCCTGGCCTCAAGTGATCCTCCCGCCTCAGCCTCCCAAAGTGCTGGGATTATAGGCATGAGCCACTGGGCTCACTCGAG

**Table S4. Multivariable Cox regression analysis of factors prognostic for patient survival.**

| **Factors** | **Univariate analysis** | |  | **Multivariate analysis** | |  |  |
| --- | --- | --- | --- | --- | --- | --- | --- |
|  | **Hazard ratio (95% CI)** | ***P* value** |  | **Hazard ratio (95% CI)** | ***P* value** |  |  |
| Age | 1.067 (1.057-1.078) | 1.24E-40 |  | 1.038 (1.027-1.050) | 4.5271E-12 |  |  |
| Gender | 0.82 (0.634-1.060) | 0.129 |  | 0.948 (0.730-1.230) | 0.686 |  |  |
| Race | 1.234 (0.753-2.022) | 0.405 |  | 1.374 (0.835-2.262) | 0.211 |  |  |
| WHO grade | 4.786 (3.919-5.843) | 2.69E-53 |  | 2.796 (2.202-3.551) | 3.26E-17 |  |  |
| High MEOX2 expression | 5.688 (4.233-7.644) | 9.60E-31 |  | 2.559 (1.789-3.660) | 2.6854E-07 |  |  |
| MEOX2 copy number variation | 0.642 (0.510-0.807) | 1.55E-04 |  | 1.372 (1.076-1.750) | 0.011 |  |  |


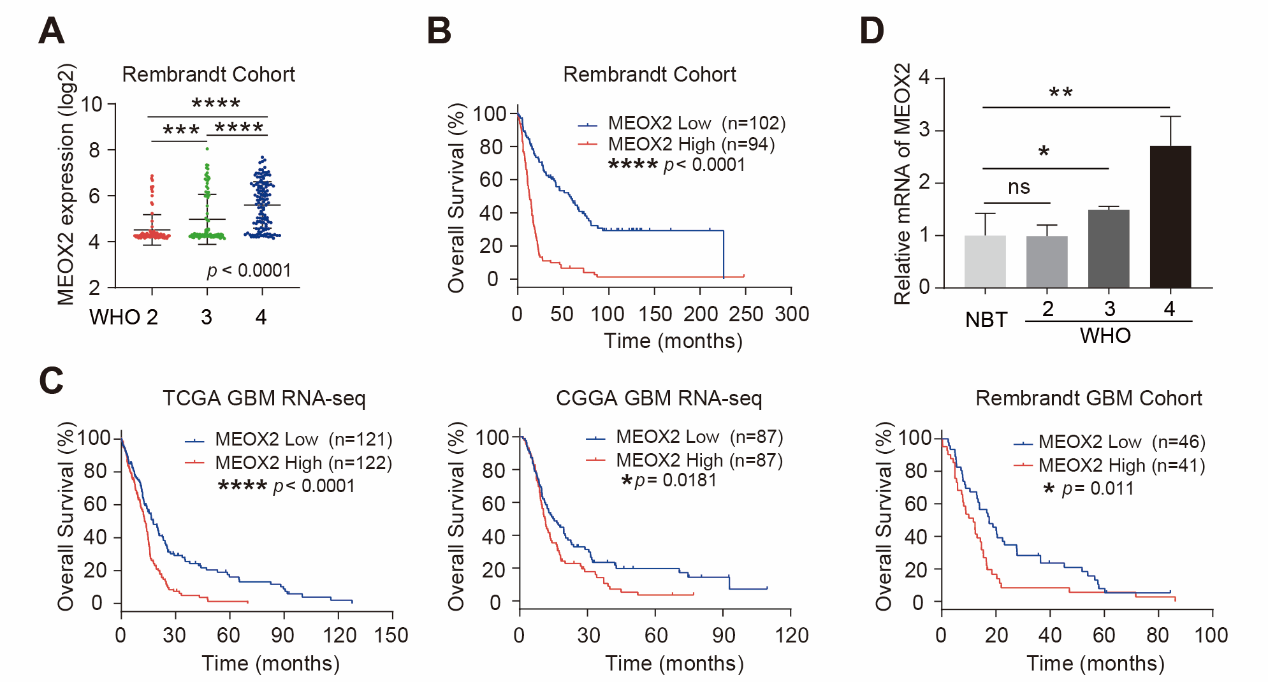


**Fig. S1 MEOX2 shows overabundance in glioma and is associated with poor prognosis. A** The mRNA expression of MEOX2 in gliomas with different WHO grades. **B** Overall survival (OS) curves of patients with high or low MEOX2 expression from Rembrandt database. **C** Overall survival (OS) curves of patients with GBM from TCGA, CGGA, Rembrandt databases stratified by MEOX2 expression. **D** RT-PCR analysis of MEOX2 mRNA expression profiling in fresh human glioma or NBT samples. ns: no significant, **p* < 0.05, ***p* < 0.01, ****p* < 0.001, *****p* < 0.0001.


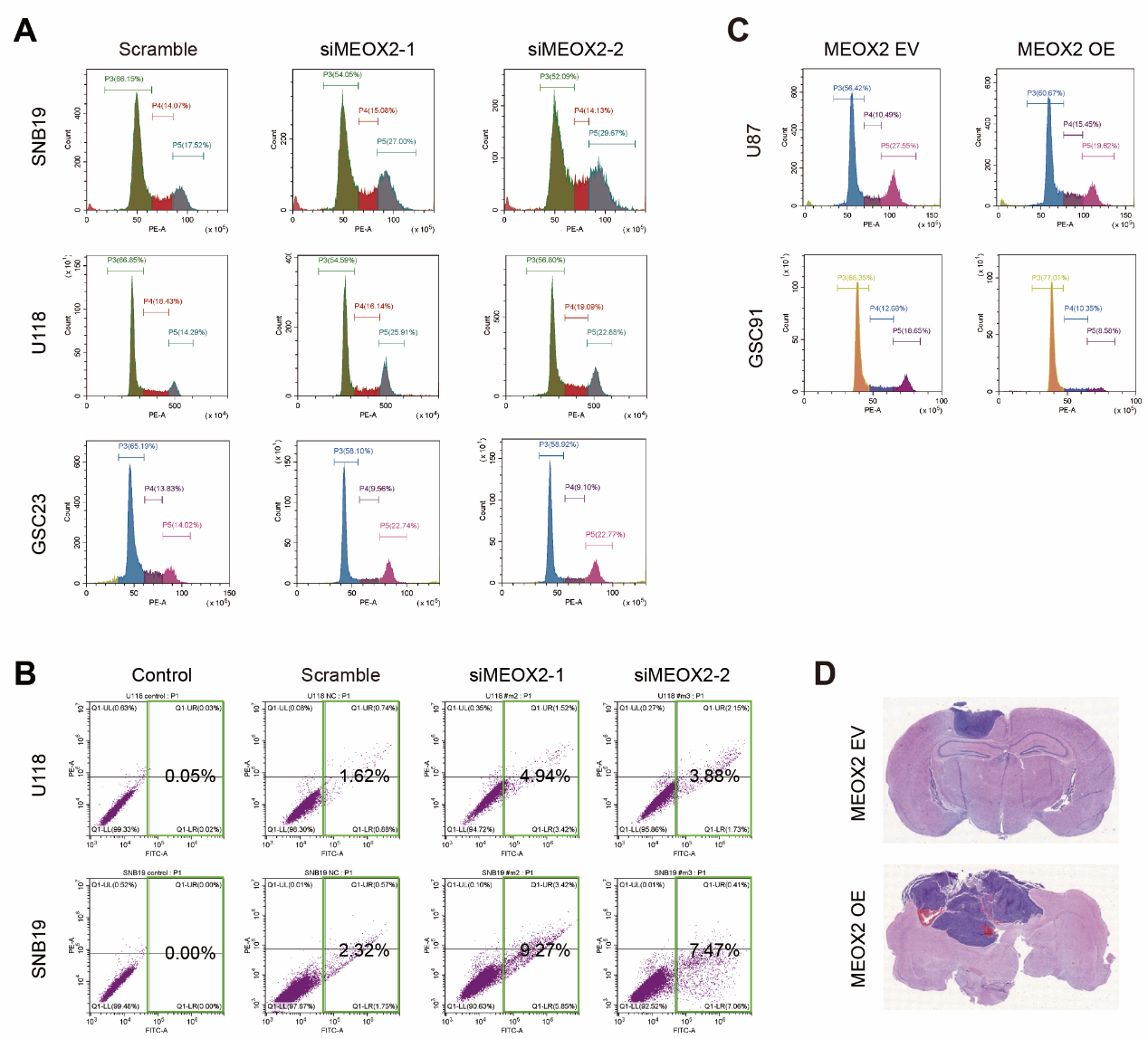


**Fig. S2** **MEOX2 mediates the proliferation of glioma cells through regulation of the cell cycle, not apoptosis. A** Cell cycle analysis of SNB19, U118 and GSC23 cells with or without MEOX2 inhibition was performed by flow cytometry. **B** Cell apoptosis analysis of SNB19 and U118 cells treated with MEOX2 siRNAs or Scramble siRNA was carried out by flow cytometry. **C** Cell cycle of U87 and GSC91 cells with or without MEOX2 overexpression was evaluated by flow cytometry. **D** MEOX2-overexpressed GSC91 cells or control cells were intracranially injected into nude mice, and representative tumor xenografts of HE staining images are shown.


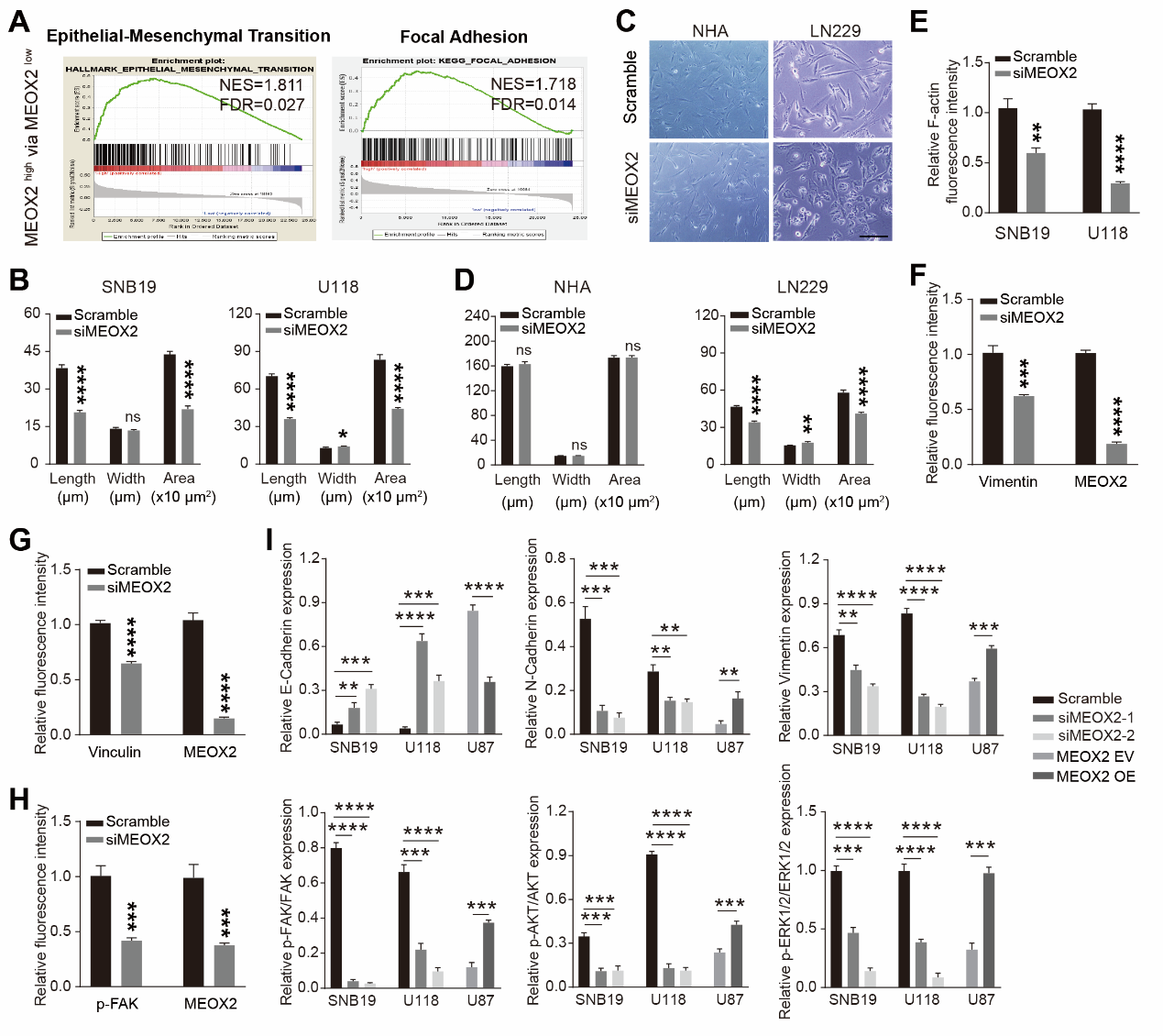


**Fig. S3 MEOX2 mediates EMT process and formation of focal adhesion in glioma cells. A** GSEA enrichment terms of EMT and focal adhesion in high MEOX2 expression vs low MEOX2 expression in CGGA gliomas. **B** Pseudopodia length, cellular width and area of SNB19 and U118 cells with or without MEOX2 knockdown were analyzed. **C** The morphology of NHA and LN229 cells with or without MEOX2 knockdown was imaged by microscope. Bar = 200 µm. **D** Pseudopodia length, cellular width and area of NHA and LN229 cells with siMEOX2 or Scramble siRNA transfection were analyzed. **E** Quantification of the relative F-actin fluorescence intensity of SNB19 and U118 cells with MEOX2 knockdown and control cells. **F**-**H** Quantification of the relative Vimentin (**F**), Vinculin (**G**) and p-FAK (Y925) (**H**) fluorescence intensity of U118 cell with or without MEOX2 inhibition. **I** Quantification of the relative protein expression of SNB19 and U118 cells with or without MEOX2 knockdown and U87 cells with or without MEOX2 overexpression. ns: no significant, **p* < 0.05, ***p* < 0.01, ****p* < 0.001, *****p* < 0.0001.


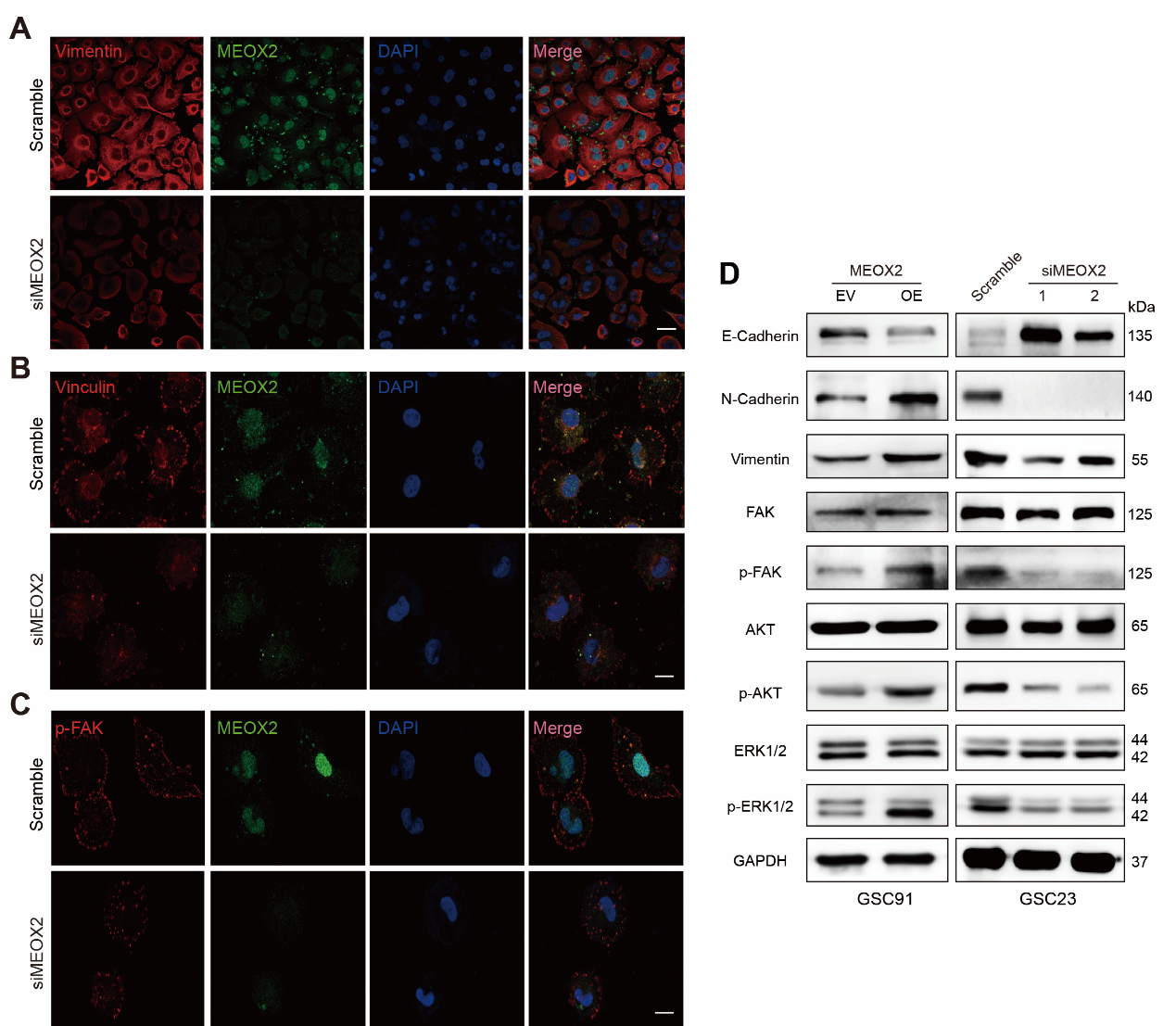


**Fig. S4 MEOX2 mediates EMT process and formation of focal adhesion in glioma cells.**

**A**-**C** Immunofluorescence staining images of SNB19 cells with MEOX2 silencing or control cells were captured by confocal microscope. Bar = 50, 100, 100 µm. **D** The protein expression of E-cadherin, N-cadherin, Vimentin, FAK, p-FAK (Y925), AKT, p-AKT (Ser473), ERK1/2 and p-ERK1/2 (Thr202/Tyr204) were analyzed by immunoblot in GSC23 cells with or without MEOX2 inhibition and GSC91 cells with or without MEOX2 overexpression.


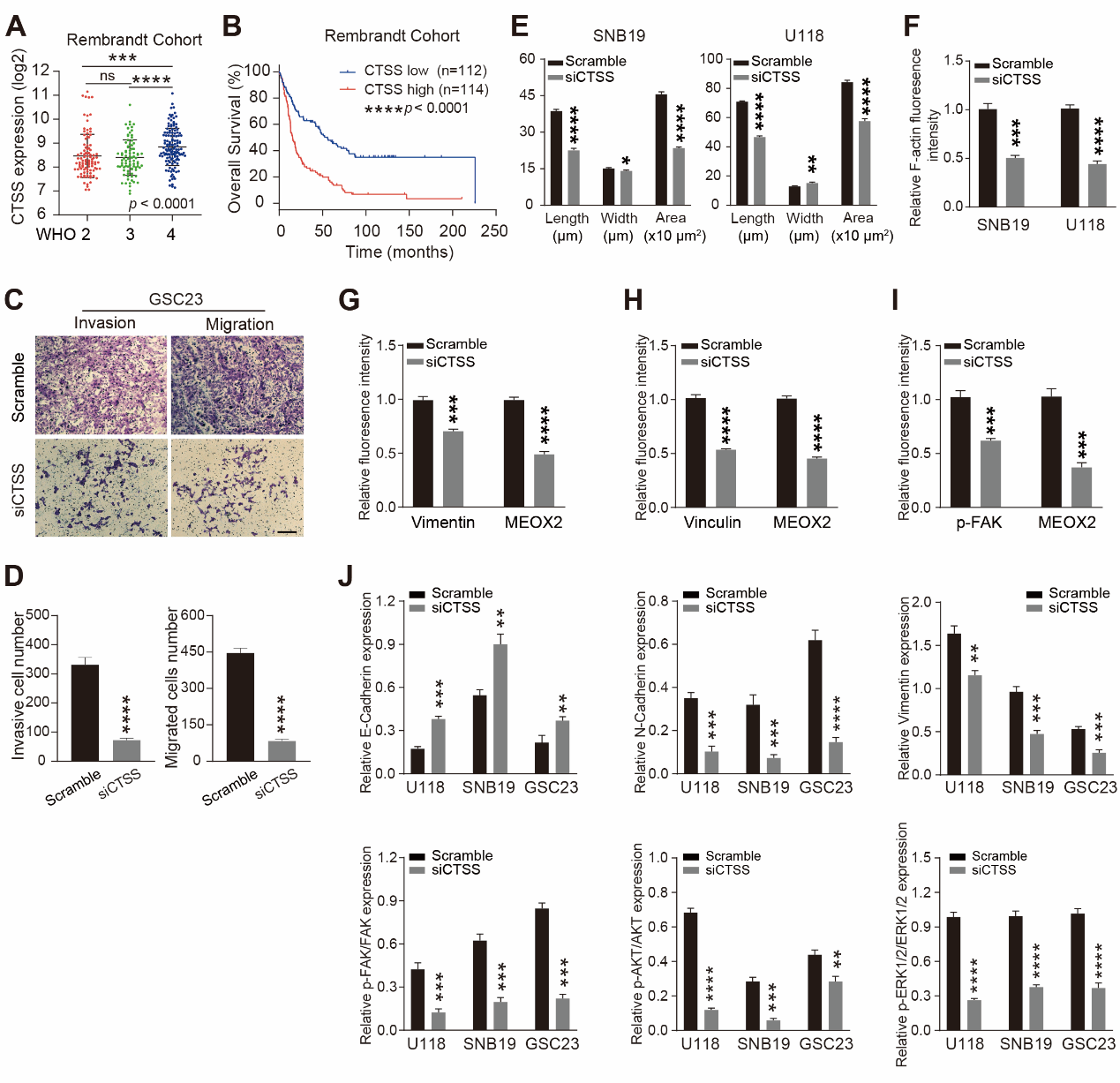


**Fig. S5** **CTSS regulates EMT process and formation of focal adhesion in glioma cells. A** The mRNA expression of CTSS in gliomas with different WHO grades from Rembrandt database. **B** Overall survival (OS) curves of patients with glioma from Rembrandt database stratified by CTSS expression. **C** Transwell assays were utilized to evaluate the invasion and migration capacities in GSC23 cells with CTSS inhibition or control cells. Bar = 100 µm. **D** Quantification of the invasive and migrated cells number. **E** Pseudopodia length, cellular width and area of SNB19 and U118 cells with or without CTSS knockdown were measured. **F** Quantification of the relative F-actin fluorescence intensity of SNB19 and U118 cells with CTSS siRNA or Scramble siRNA transfection. **G**-**I** Quantification of the relative Vimentin (**G**), Vinculin (**H**) and p-FAK (Y925) (**I**) fluorescence intensity of U118 cell with or without MEOX2 silencing. **J** Quantification of the relative protein expression of SNB19, U118 and GSC23 cells with or without CTSS inhibition. ns: no significant, **p* < 0.05, ***p* < 0.01, ****p* < 0.001, *****p* < 0.0001.


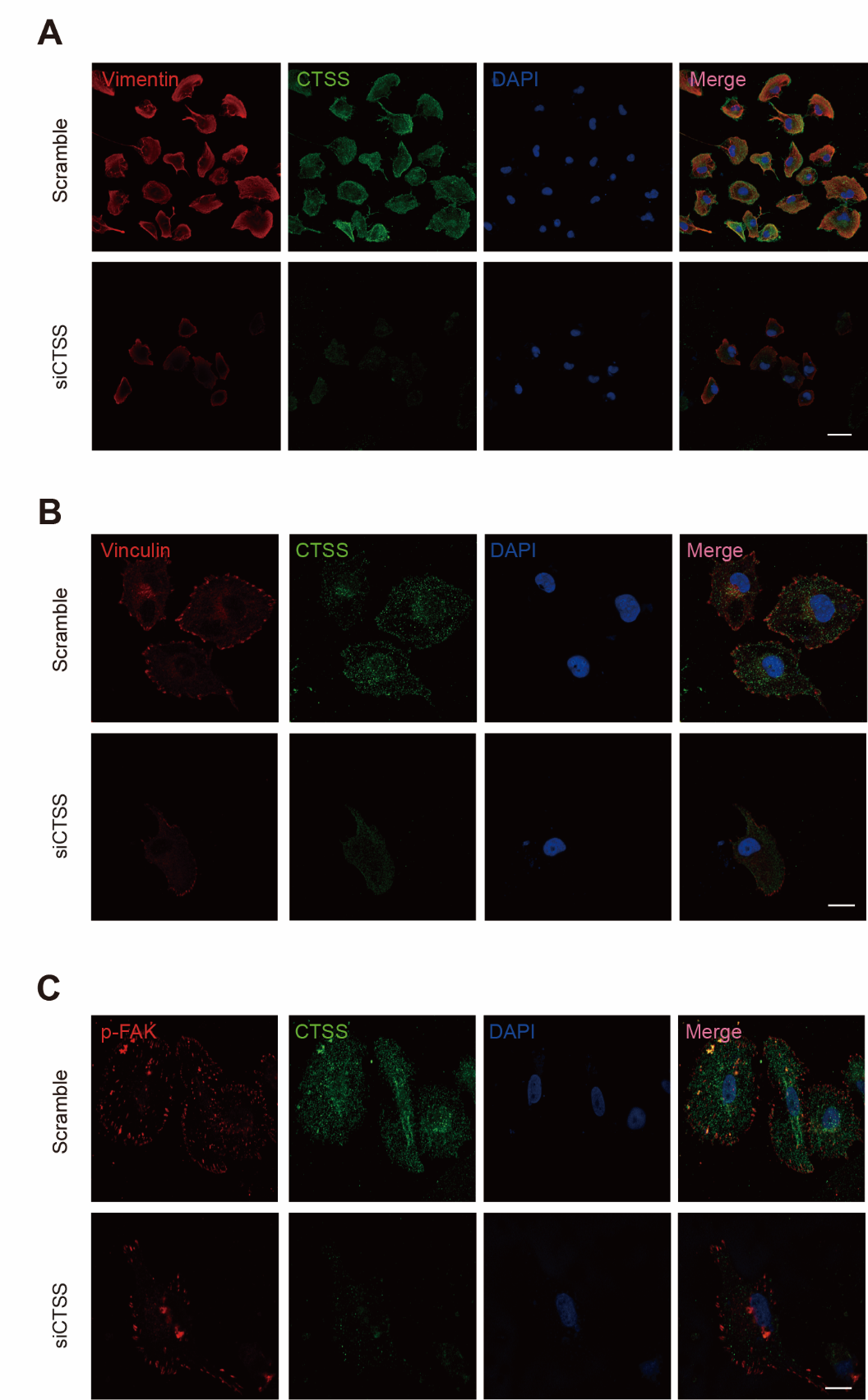


**Fig. S6** **CTSS regulates EMT process and formation of focal adhesion in glioma cells. A**-**C** Vimentin (**A**), Vinculin (**B**) and p-FAK (Y925) (**C**) immunofluorescence staining of SNB19 cells with CTSS knockdown or control cells were observed by confocal microscope. Bar = 40, 20, 20 µm.


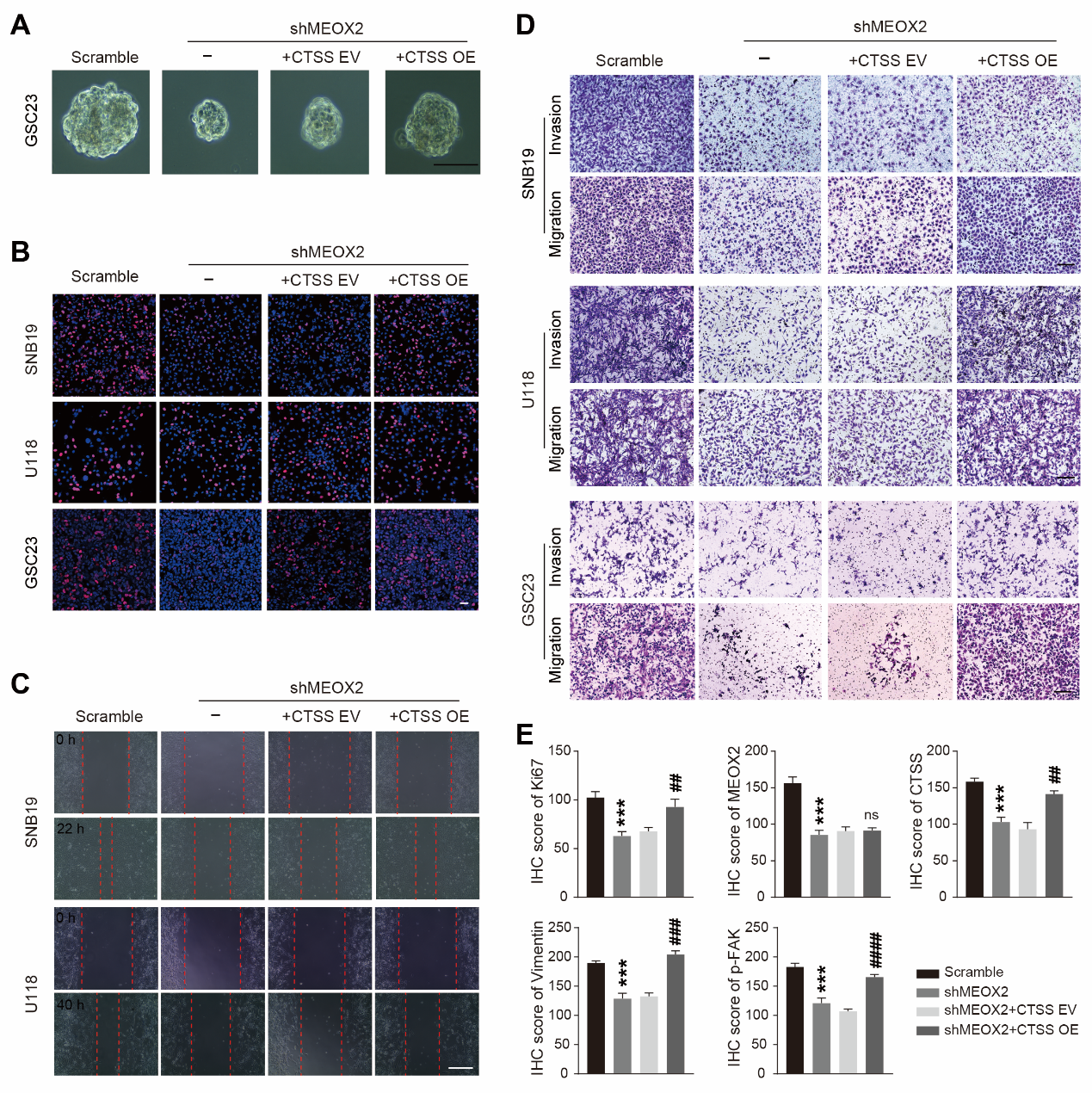


**Fig. S7 CTSS re-expression partly abrogates cell proliferation and motility inhibition caused by MEOX2 silencing in glioma. A** Representative sphere images of GSC23 cells treated with Scramble shRNA, shMEOX2, shMEOX2 + CTSS EV and shMEOX2 + CTSS OE. Bar = 100 µm. **B** Representative EdU immunofluorescence staining images of SNB19, U118 and GSC23 cells infected with Scramble shRNA, shMEOX2, shMEOX2 + CTSS EV and shMEOX2 + CTSS OE. Bar = 50 µm. **C** Cell migration ability of SNB19 and U118 cells treated with Scramble shRNA, shMEOX2, shMEOX2 + CTSS EV and shMEOX2 + CTSS OE was evaluated by wound healing assays. Bar = 400 µm. **D** Cell invasion and migration capacities of SNB19, U118 and GSC23 cells infected with Scramble shRNA, shMEOX2, shMEOX2 + CTSS EV and shMEOX2 + CTSS OE was assessed by transwell assays. Bar = 200 µm. **E** Quantification of the IHC score of Ki67, MEOX2, CTSS, Vimentin and p-FAK. * shMEOX2 group vs Scramble group, ****p* < 0.001; # shMEOX2+CTSS OE group vs shMEOX2+CTSS EV group, ns: no significant, ##*p* < 0.01, ###*p* < 0.001, ####*p* < 0.0001.

**Original western blot images**


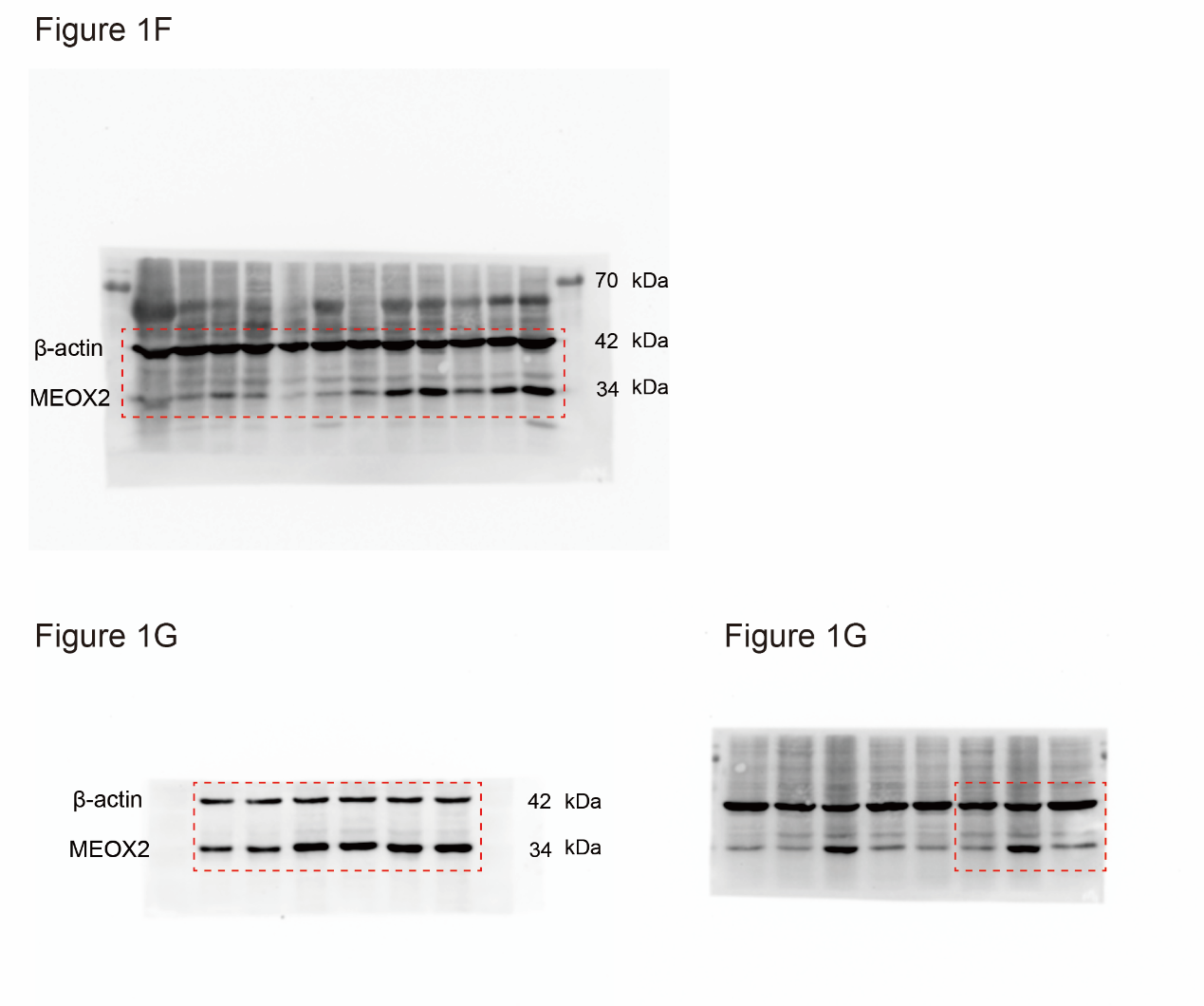


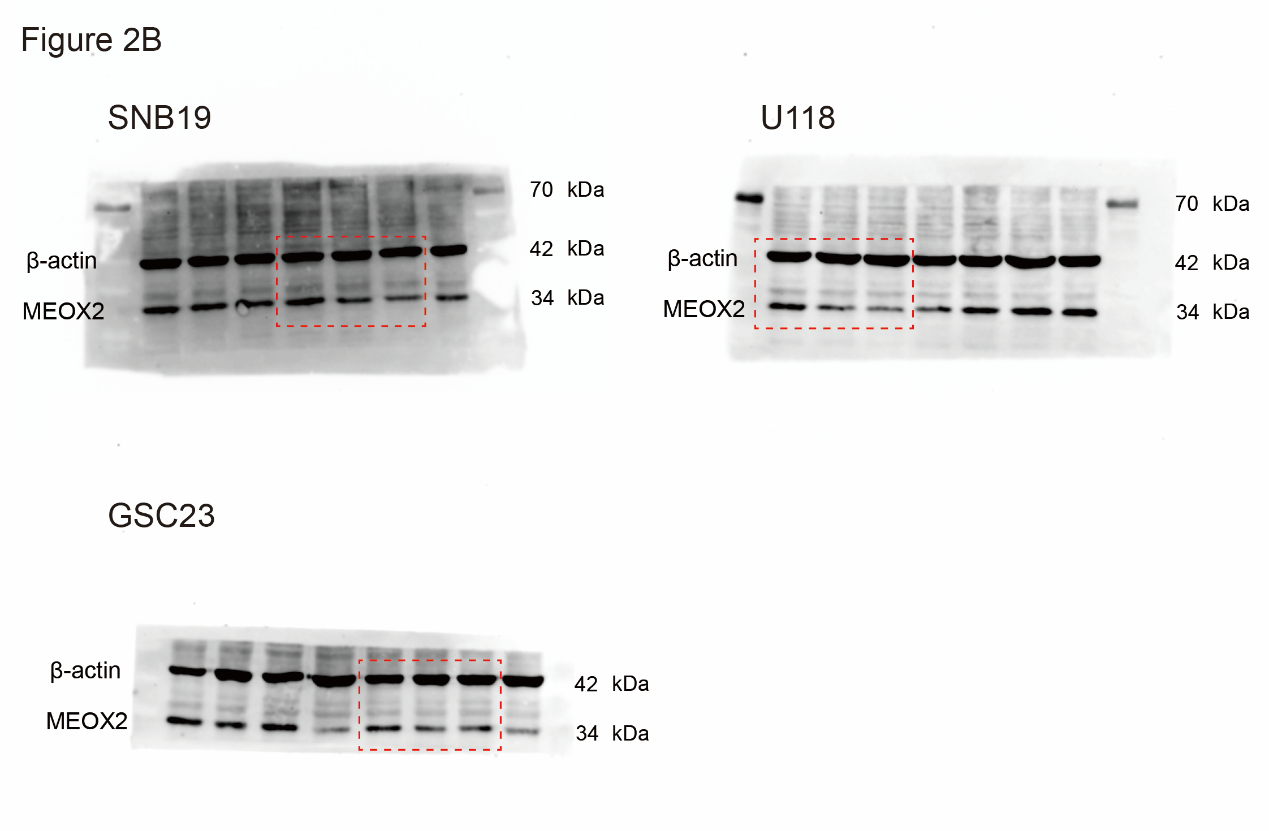

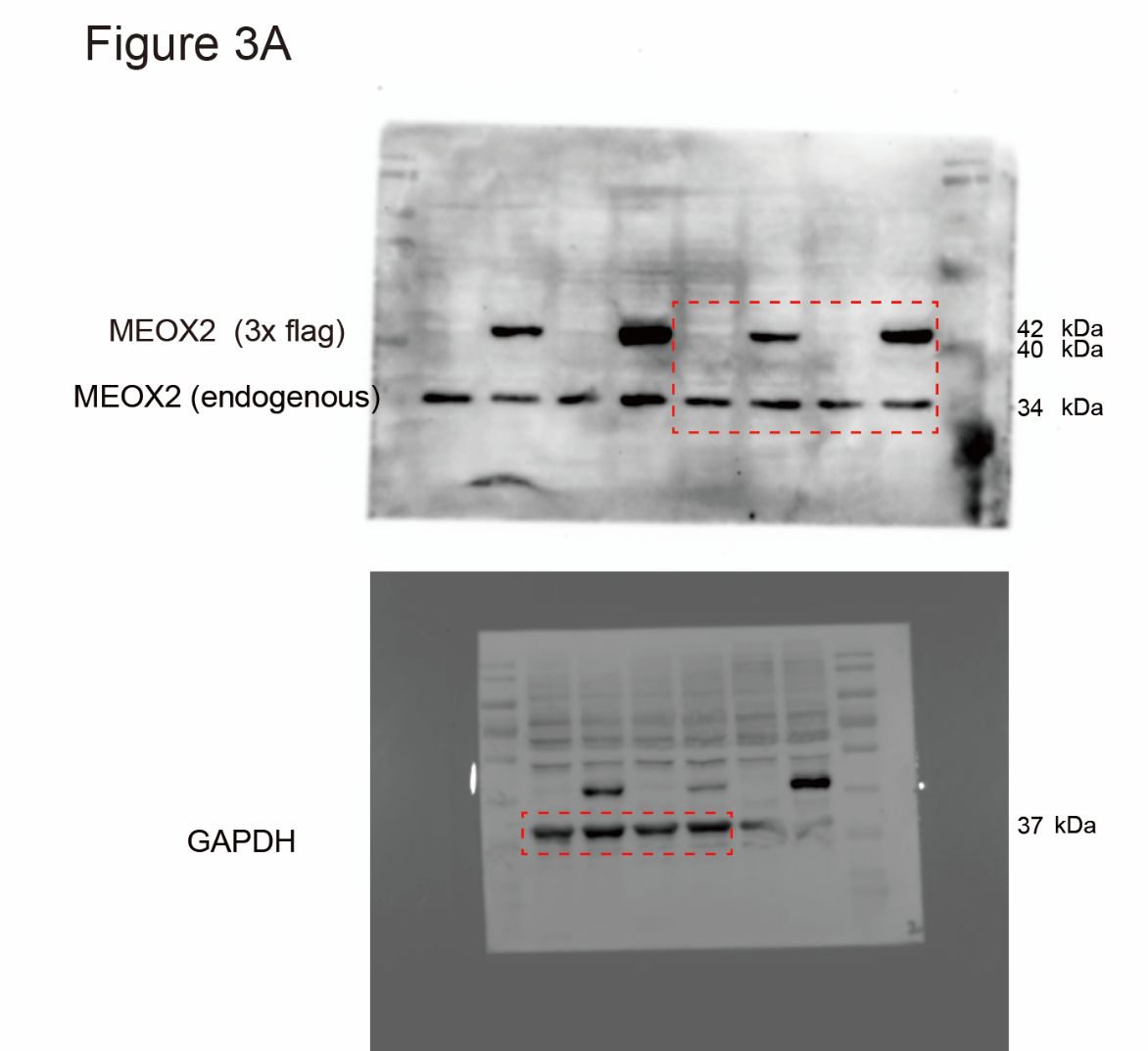

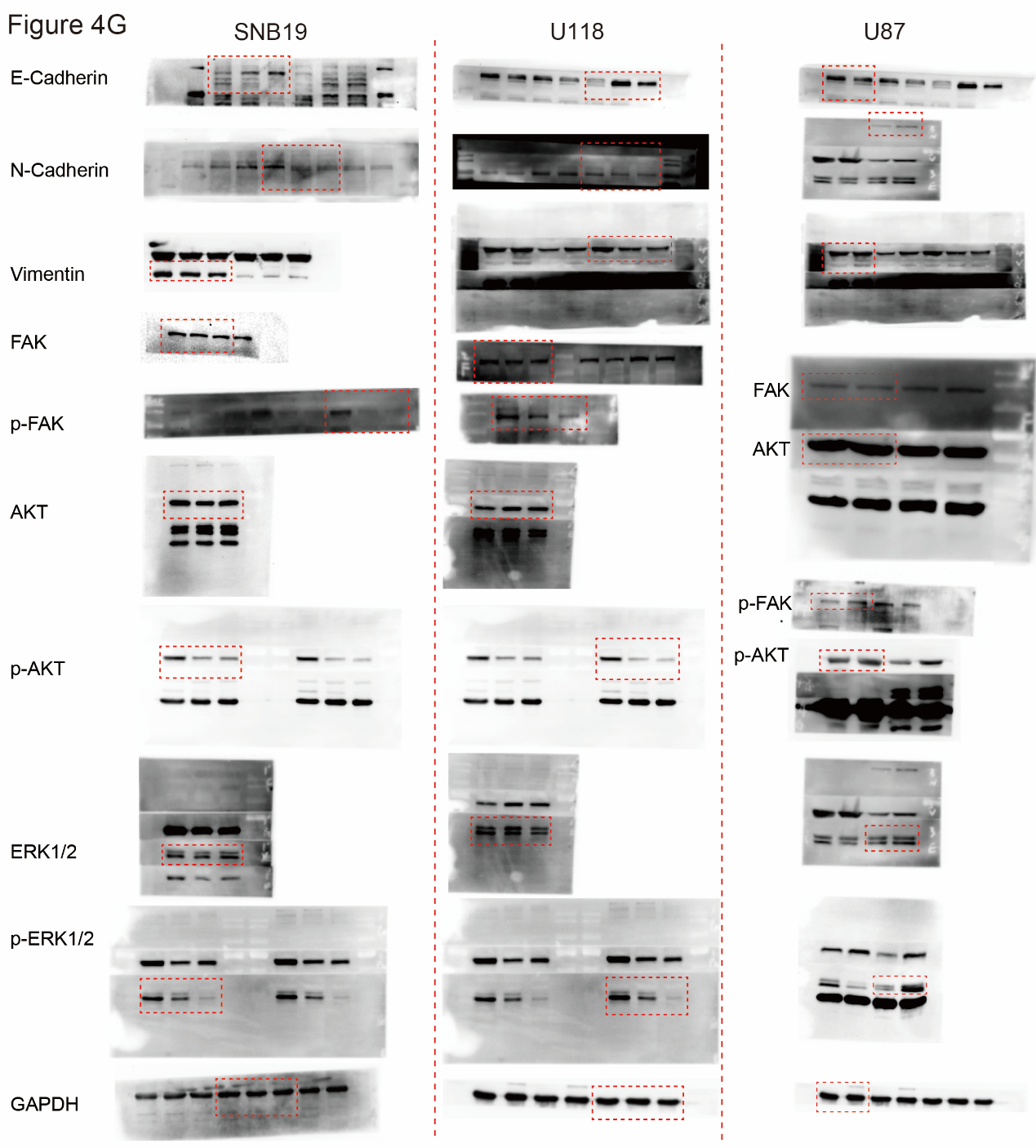

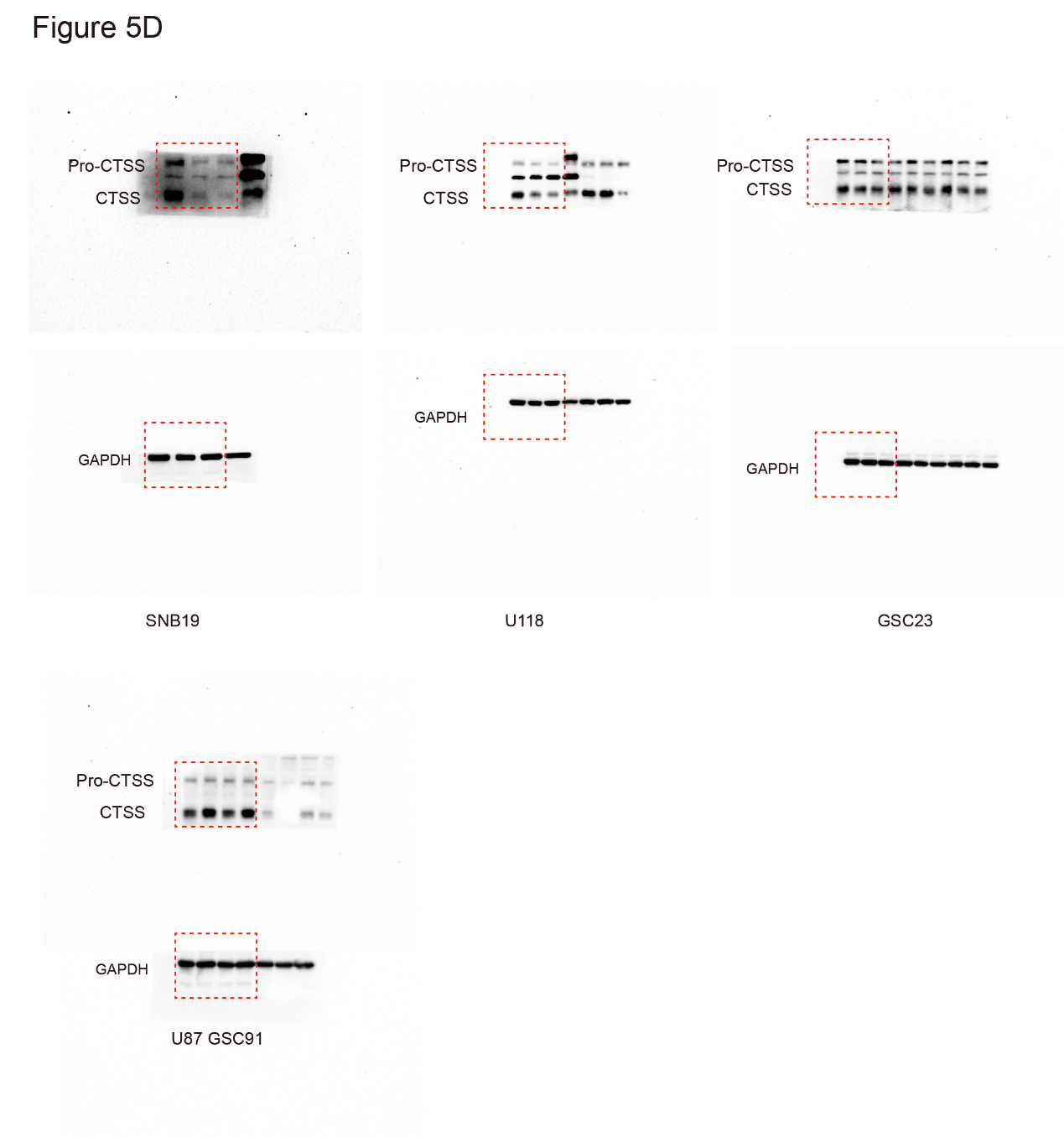

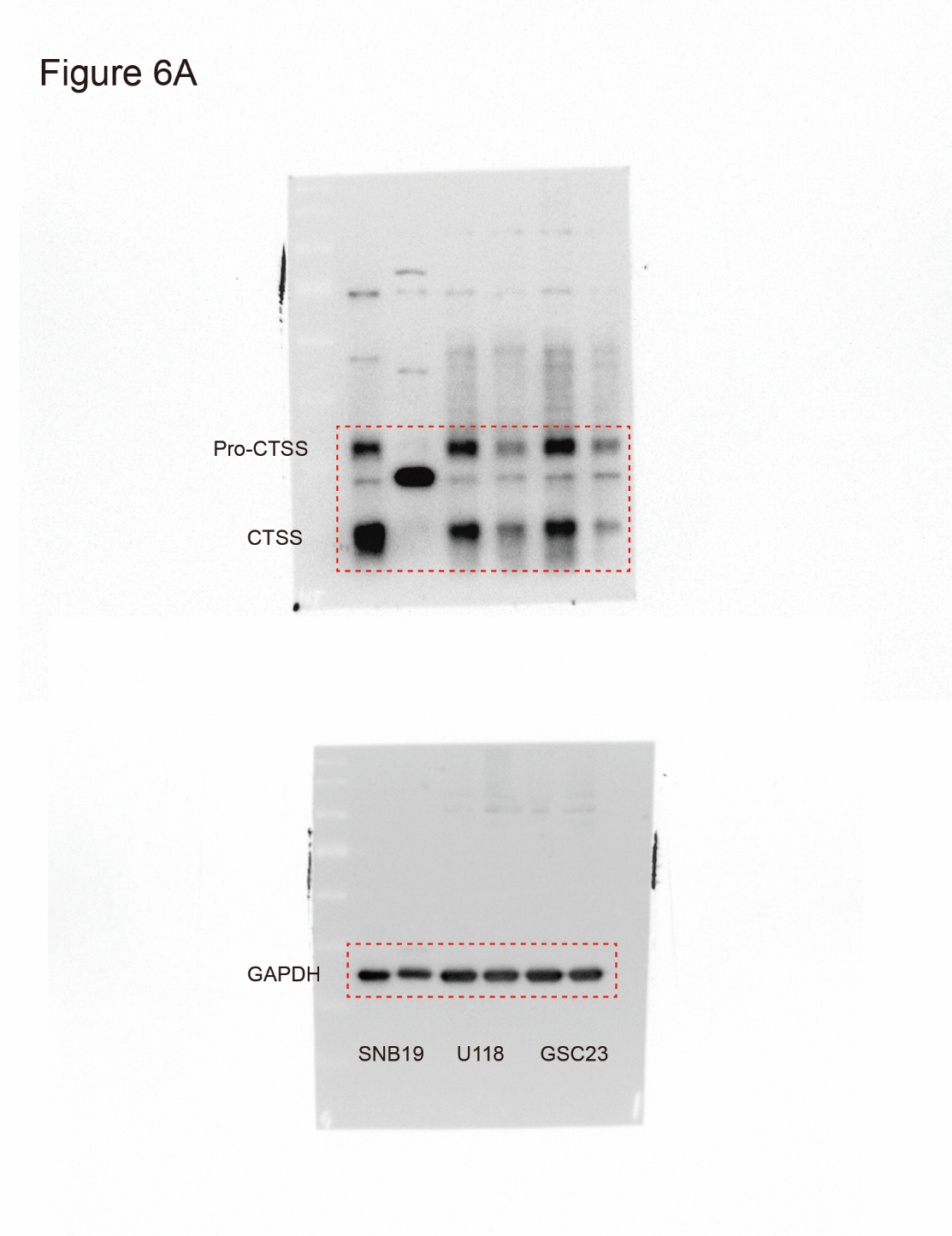

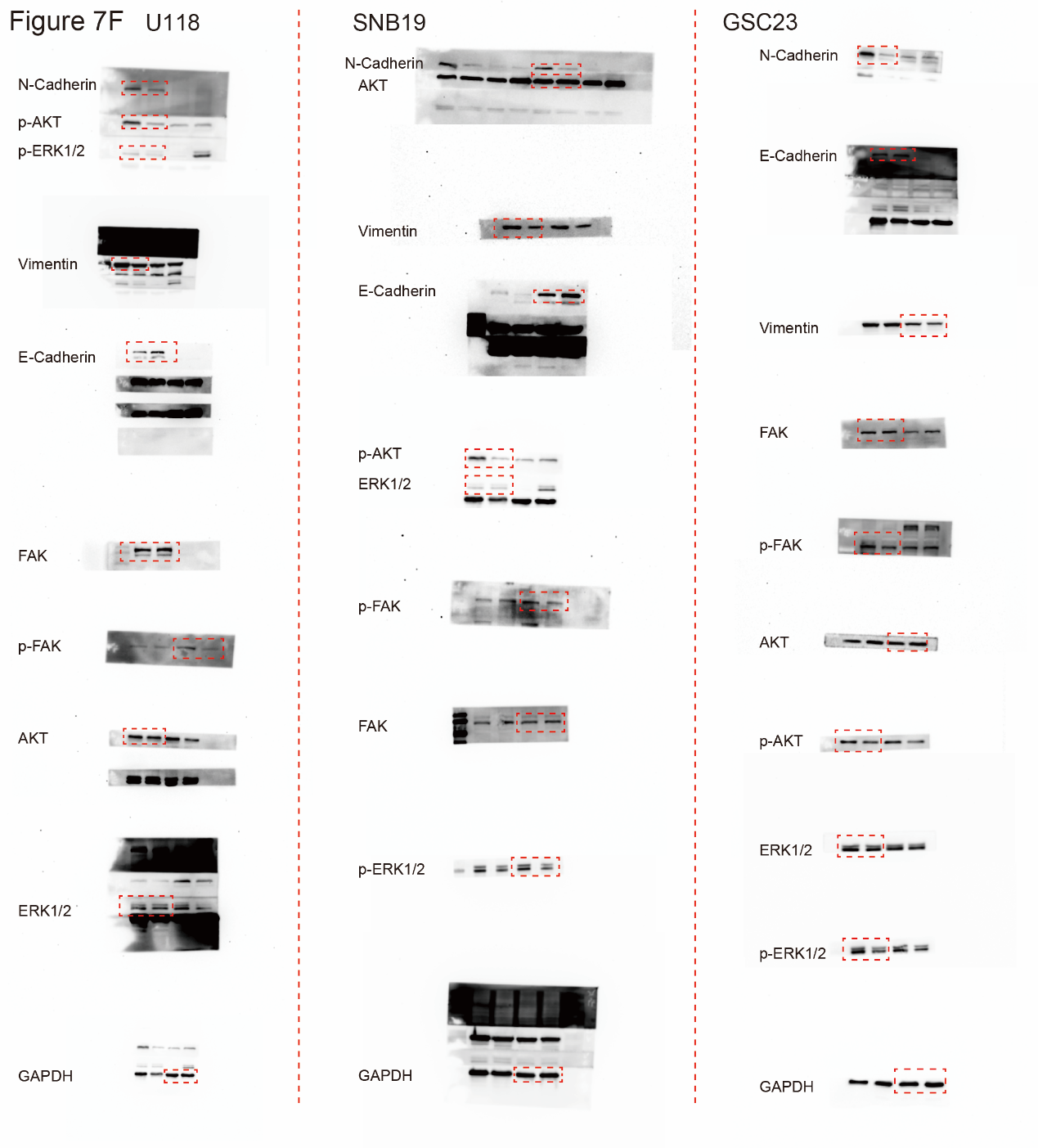

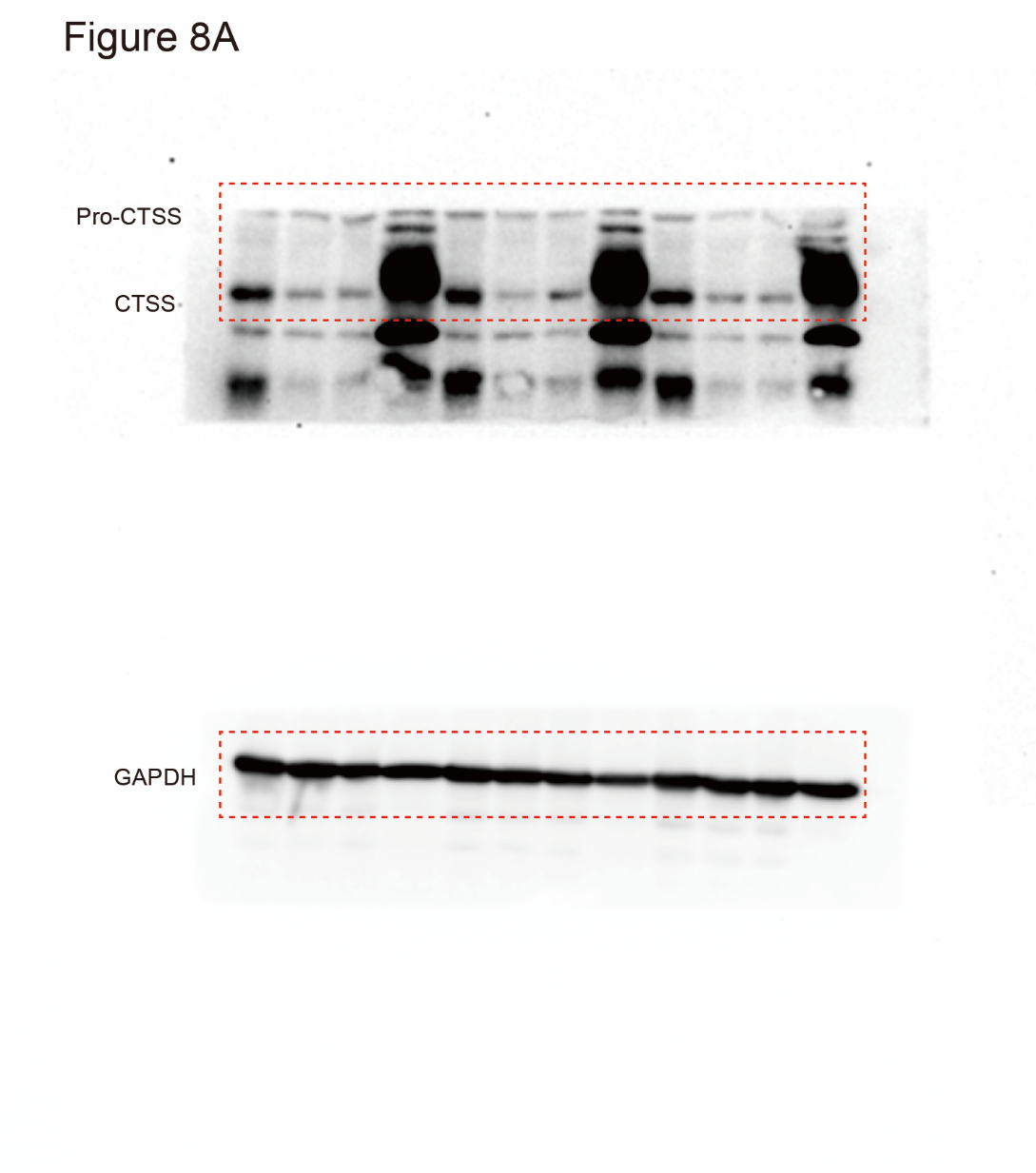

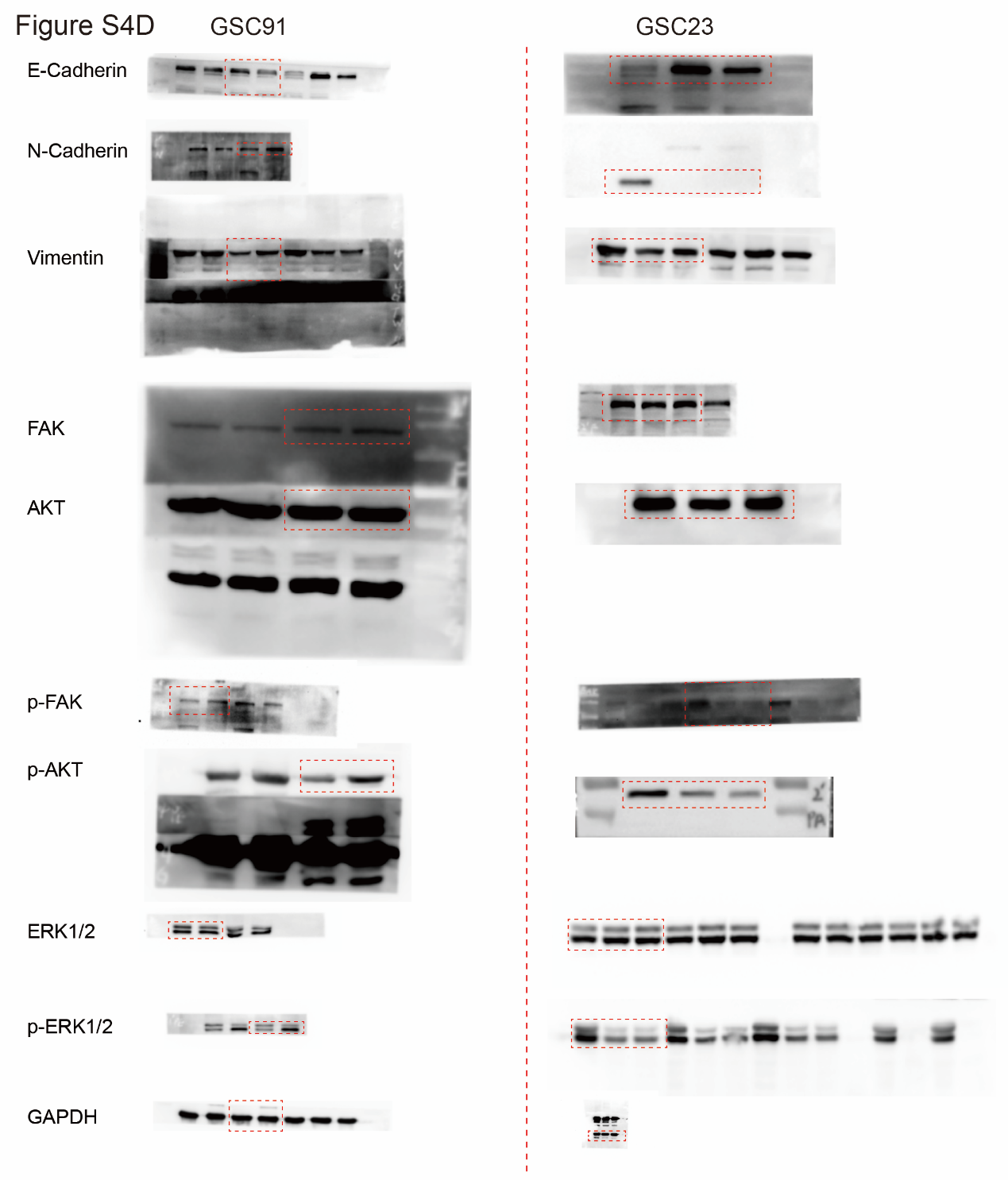

Supplement: Supplementary file 1 — Supplementary Materials [file 41419_2022_4845_MOESM1_ESM.docx]
